# Tracing HIV-1 transmission: envelope traits of HIV-1 transmitter and recipient pairs

**DOI:** 10.1186/s12977-016-0299-0

**Published:** 2016-09-05

**Authors:** Corinna S. Oberle, Beda Joos, Peter Rusert, Nottania K. Campbell, David Beauparlant, Herbert Kuster, Jacqueline Weber, Corinne D. Schenkel, Alexandra U. Scherrer, Carsten Magnus, Roger Kouyos, Philip Rieder, Barbara Niederöst, Dominique L. Braun, Jovan Pavlovic, Jürg Böni, Sabine Yerly, Thomas Klimkait, Vincent Aubert, Alexandra Trkola, Karin J. Metzner, Huldrych F. Günthard, The Swiss HIV Cohort Study (SHCS), V Aubert, M Battegay, E Bernasconi, J Böni, DL Braun, HC Bucher, C Burton-Jeangros, A Calmy, M Cavassini, G Dollenmaier, M Egger, L Elzi, J Fehr, J Fellay, H Furrer, CA Fux, M Gorgievski, H Günthard, D Haerry, B Hasse, HH Hirsch, M Hoffmann, I Hösli, C Kahlert, L Kaiser, O Keiser, T Klimkait, R Kouyos, H Kovari, B Ledergerber, G Martinetti, B Martinez de Tejada, C Marzolini, K Metzner, N Müller, D Nadal, D Nicca, G Pantaleo, A Rauch, S Regenass, C Rudin, F Schöni-Affolter, P Schmid, R Speck, M Stöckle, P Tarr, A Trkola, P Vernazza, R Weber, S Yerly

**Affiliations:** 1 Division of Infectious Diseases and Hospital Epidemiology, University Hospital Zurich, University of Zurich, Zurich, Switzerland; 2 Institute of Medical Virology, University of Zurich, Zurich, Switzerland; 3 Laboratory of Virology, University Hospital Geneva, University of Geneva, Geneva, Switzerland; 4 Department of Biomedicine, University of Basel, Basel, Switzerland; 5 Division of Immunology and Allergy, University Hospital Lausanne, University of Lausanne, Lausanne, Switzerland

**Keywords:** HIV-1, Transmission, Envelope, Neutralization, Replicative capacity, IFNα, Entry

## Abstract

**Background:**

Mucosal HIV-1 transmission predominantly results in a single transmitted/founder (T/F) virus establishing infection in the new host despite the generally high genetic diversity of the transmitter virus population. To what extent HIV-1 transmission is a stochastic process or driven by selective forces that allow T/F viruses best to overcome bottlenecks in transmission has not been conclusively resolved. Building on prior investigations that suggest HIV-1 envelope (Env) features to contribute in the selection process during transmission, we compared phenotypic virus characteristics of nine HIV-1 subtype B transmission pairs, six men who have sex with men and three male-to-female transmission pairs.

**Results:**

All recipients were identified early in acute infection and harbored based on extensive sequencing analysis a single T/F virus allowing a controlled analysis of virus properties in matched transmission pairs. Recipient and transmitter viruses from the closest time point to transmission showed no signs of selection for specific Env modifications such as variable loop length and glycosylation. Recipient viruses were resistant to circulating plasma antibodies of the transmitter and also showed no altered sensitivity to a large panel of entry inhibitors and neutralizing antibodies. The recipient virus did not consistently differ from the transmitter virus in terms of entry kinetics, cell–cell transmission and replicative capacity in primary cells. Our paired analysis revealed a higher sensitivity of several recipient virus isolates to interferon-α (IFNα) which suggests that resistance to IFNα cannot be a general driving force in T/F establishment.

**Conclusions:**

With the exception of increased IFNα sensitivity, none of the phenotypic virus properties we investigated clearly distinguished T/F viruses from their matched transmitter viruses supporting the notion that at least in subtype B infection HIV-1 transmission is to a considerable extent stochastic.

**Electronic supplementary material:**

The online version of this article (doi:10.1186/s12977-016-0299-0) contains supplementary material, which is available to authorized users.

## Background

The identification of host and viral determinants that govern human immunodeficiency virus type 1 (HIV-1) transmission and infection is essential for the development of a targeted prevention approach. The observation that virus populations in acute infection are very homogeneous [1–4] contrasting the high diversity of the HIV-1 quasispecies observed in chronic infection [5] suggested that HIV-1 encounters a population bottleneck upon transmission (reviewed in [6, 7]). This was further corroborated by the discovery that the majority of transmitted viruses are CCR5 utilizing (R5 tropic) [1, 8, 9], although later during natural history of the infection a tropism switch to CXCR4 co-receptor utilizing (X4 tropic) HIV-1 variants occurs in untreated infection in approximately 50 % of individuals [10]. Early findings that only few viral variants are transmitted and seed a new infection were substantiated by single genome amplification (SGA) approaches which revealed that depending on the mode of sexual transmission, in 60–90 % of cases a single HIV-1 variant [termed the transmitted/founder (T/F) virus], establishes an infection [11–15]. These findings funneled intensive research efforts aiming to understand if the limited transmission of viral variants is due to selective forces acting during transmission and early infection or is simply the result of a stochastic process (reviewed in [6, 7]).

A potential influence of genotypic and phenotypic traits of the HIV-1 envelope (Env) in steering transmission was first suggested in a study of eight heterosexual HIV-1 subtype C transmission pairs [16]. Recipient Envs had shorter V1–V4 loops with less potential N-linked glycosylation sites (PNGS) and showed increased sensitivity to the corresponding transmitter’s plasma. Reduced length and glycosylation of Env proved a trait in HIV-1 subtype A, C and D, and possibly also in subtype B transmission [17–23]. Less clear is the influence of neutralization sensitivity on transmission and variable patterns of T/F virus sensitivity to monoclonal antibodies and transmitter’s plasma were observed in subtype B and C infection [11, 19, 20, 24–26]. T/F viruses proved equally efficient as corresponding transmitter or chronic control viruses in establishing CD4 and CCR5 receptor interactions [20, 24, 27–29] and macrophage tropism appears not to be associated with transmission as T/F viruses efficiently infect CD4^+^ T-cells but not monocyte-derived macrophages (MDMs) [28, 30, 31]. Furthermore, acute Envs have been suggested to bind the integrin α4β7 with high affinity [29, 32, 33]. T/F infectious molecular clones of HIV-1 subtype B and C were, when compared to chronic control viruses more infectious, harbored more Env, and bound to dendritic cells more efficiently [34]. Additionally, T/F viruses were reported to be more resistant to interferon-α (IFNα) as compared to chronic stage viruses from the same or control individuals raising the possibility that IFNα resistance may aid these viruses to evade early immune responses [34, 35]. Interestingly, a study in injection drug users of HIV-1 subtype B as well as a recent publication investigating six HIV-1 subtype C transmission pairs could not reproduce higher IFNα resistance of acute viruses [26, 36].

While CCR5 tropism is clearly selected for in transmission, not all viral properties described as selective parameters during transmission are consistently linked with T/F viruses across different studies. This may in part be due to differences in the population bottleneck that might vary depending on the HIV-1 subtype and the transmission mode studied. Differences between the transmitter and T/F viruses may also be very subtle and their identification may thus depend on studying defined transmission pairs where viruses were derived at time points very close to the transmission date. Not all studies on the in vitro phenotyping of T/F viruses described to date had access to the optimal transmitter viruses: Often transmitters are not known, which restricted the comparison to unrelated chronic control viruses. Considering the high variability of the HIV-1 Env and that minor sequence changes can have a considerable impact on Env functionality cross-sectional analysis alone may not suffice to define selective forces of transmission. We therefore thought it crucial to perform a paired analysis of confirmed transmission pairs to investigate the influence of Env traits in the transmission process. We investigated in total nine HIV-1 subtype B transmission pairs that we phylogenetically identified within the Zurich Primary HIV Infection (ZPHI) study and the Swiss HIV Cohort Study (SHCS). In six pairs HIV was acquired through men who have sex with men (MSM) transmission and in three pairs through male-to-female (MTF) transmission. We analyzed phenotypic properties of transmitter and recipient viruses with a particular focus on Env to define if T/F viruses harbor distinct features that are crucial during the earliest stages of HIV-1 infection endowing viruses with a transmission advantage. Based on the investigated phenotypic virus properties, our findings suggest that at least in subtype B infection HIV-1 transmission is to a considerable extent stochastic.

## Results

### Large-scale phylogenetic analysis of *polymerase* sequences in two Swiss HIV cohorts identifies linked transmission pairs

A paired analysis of viruses from confirmed transmission pairs is key to understand the selective forces in HIV-1 transmission. To identify transmission pairs amongst individuals enrolled in the ZPHI study and the SHCS we utilized *polymerase* (*pol*) sequences derived from genotypic resistance tests in a phylogenetic analysis [37]. The ZPHI study is a prospective, observational, single center cohort enrolling patients with a confirmed acute or recent primary HIV-1 infection [37–39]. The SHCS is a nationwide cohort incorporating all HIV-1 infected adults living in Switzerland and includes at least 53 % of all HIV infections ever diagnosed in Switzerland [40, 41].

By subjecting 508 *pol* sequences from 300 ZPHI patients and 23,705 *pol* sequences from over 19,000 SHCS patients to phylogenetic analysis we were able to identify probable transmission pairs. Pairs with a genetic distance in *pol* of <1.5 % (Additional file 1: Figure S1a) were further examined [37]. We defined the estimated date of transmission (EDT) by incorporating available information of recipients on previous HIV tests, Western blot results, avidity assays, the start of acute retroviral symptoms and potential risk situations [37–39]. Additionally, we took clinical and epidemiological data of potential transmitters at the EDT such as viral load, antiretroviral treatment and risk group into account for determining transmission pairs and of three pairs, transmitters disclosed that they had infected corresponding recipients. To confirm virus transmission, we selected transmitter and recipient plasma from the biobanks of the ZPHI and the SHCS from the closest possible time point to transmission to perform SGA of full-length *env*. Of note, although a greater number of transmission pairs was identified with this iterative analysis involving phylogenetic data of *pol* and *env* and the available patients’ history, we focused here on studying nine HIV-1 subtype B transmission pairs (transmitter T8 is a subtype B/F1 recombinant) as for these bio bank samples for follow-up experiments were available. Of the nine transmission pairs studied, six recipients acquired HIV-1 via MSM and three recipients via MTF transmission. In total, 174 SGA *env* sequences of transmitters and recipients from those nine transmission pairs were derived and used to confirm transmission pair linkage by *env* phylogenetic analysis and to define T/F populations in the assumed recipients (Additional file 1: Figure S1b). The recipients were identified and sampled after a median duration of 49 days (range 26–90 days) after EDT confirming the status of early infection (Table 1). Available samples of transmitters were within a median time interval of 57 days of the EDT (range −20 to 170 days; Table 1; Additional file 2: Figure S2). Four out of the nine transmitters had a relatively low viral diversity (*env* diversity < 1 %). One of these individuals was recently infected and two others had started antiretroviral treatment immediately after infection and transmitted HIV-1 upon virus rebound after structured treatment interruption (Table 2). As most prior studies focused on high diversity transmission we considered it important to include low diversity cases as well in our study as acutely infected transmitters account for a large proportion of new infections [42–44]. Furthermore, although high virus diversity will provide more opportunity for selection processes, low diversity transmission pairs where transmitter and recipient have high sequence similarity may allow more ready detection of genotypes and phenotypes that develop early after infection and which are essential for transmission.

**Table 1:** Patients’ and virus’ characteristics of HIV-1 subtype B infected transmission pairs

Pair	Transmission mode	Virus tropism		Interval (days)^a^		Viral load^b^		CD4 count^c^	
		T	R	T	R	T	R	T	R
T1–R1	MSM	R5	R5	95	32	153,000	2,325,000	287	273
T2–R2	HET (MTF)	R5	R5	49	42	18,300	412,857	416	625
T3–R3	MSM	R5X4	R5	170	84	34,600	14,400	115	656
T4–R4	HET (MTF)	R5	R5	57	43	9813	66,000	681	346
T5–R5	MSM	R5	R5	−20	26	43,200	373,000	422	430
T6–R6	HET (MTF)	R5	R5	63	49	257,532	428,000	623	170
T7–R7	MSM	R5	R5	33	61	2830	5,970,000	595	362
T8–R8	MSM	R5	R5	3	56	2260	56,300	664	564
T9–R9	MSM	R5	R5	89	90	27,800	1550	429	516

*T* transmitter, *R* recipient, *MSM* men who have sex with men, *HET* heterosexual, *MTF* male-to-female

^a^Time from the EDT to the day of sample collection. Negative value means sample was collected before EDT

^b^HIV-1 RNA copies/ml of plasma at the day of sample collection

^c^CD4^+^ T cells/μl at the day of sample collection

**Table 2:** Pairwise *pol* distance, *env* population distance and diversity along with infection stage of transmission pairs

Pair	*pol* distance (%)	No. of *env* sequences		*env* distance (%)	*env* diversity (%)		Infection stage	
		T	R		T	R	T	R
T1–R1	0.24	28	23	1.45	3.05	0.26	Chronic	Acute
T2–R2	0.48	21	27	2.45	4.61	0.18	Chronic	Acute
T3–R3	0.71	20	11	1.49	3.37	0.10	Chronic	Acute
T4–R4	0.48	30	10	0.85	1.69	0.07	Chronic	Acute
T5–R5	0.23	18	19	0.28	0.93	0.22	Chronic	Acute
T6–R6	0.24	25	18	0.11	0.51	0.34	Recent	Acute
T7–R7	0.15	5	18	0.12	0.34	0.12	eART-STI^b^	Acute
T8–R8	0.08	17	19	0.32	0.50	0.11	eART-STI^b^	Acute
T9–R9	0.16	5^a^	8	1.40	1.08	0.35	Recent	Acute

*T* transmitter, *R* recipient

^a^Sequences of T9 are derived from full-length *env* clones only after several SGA attempts failed

^b^ Early antiretroviral treatment (eART) was started immediately after infection and HIV-1 was transmitted upon virus rebound after structured treatment interruption (STI)

### Sequence characteristics of transmitter and recipient viruses

**Fig. 1:**
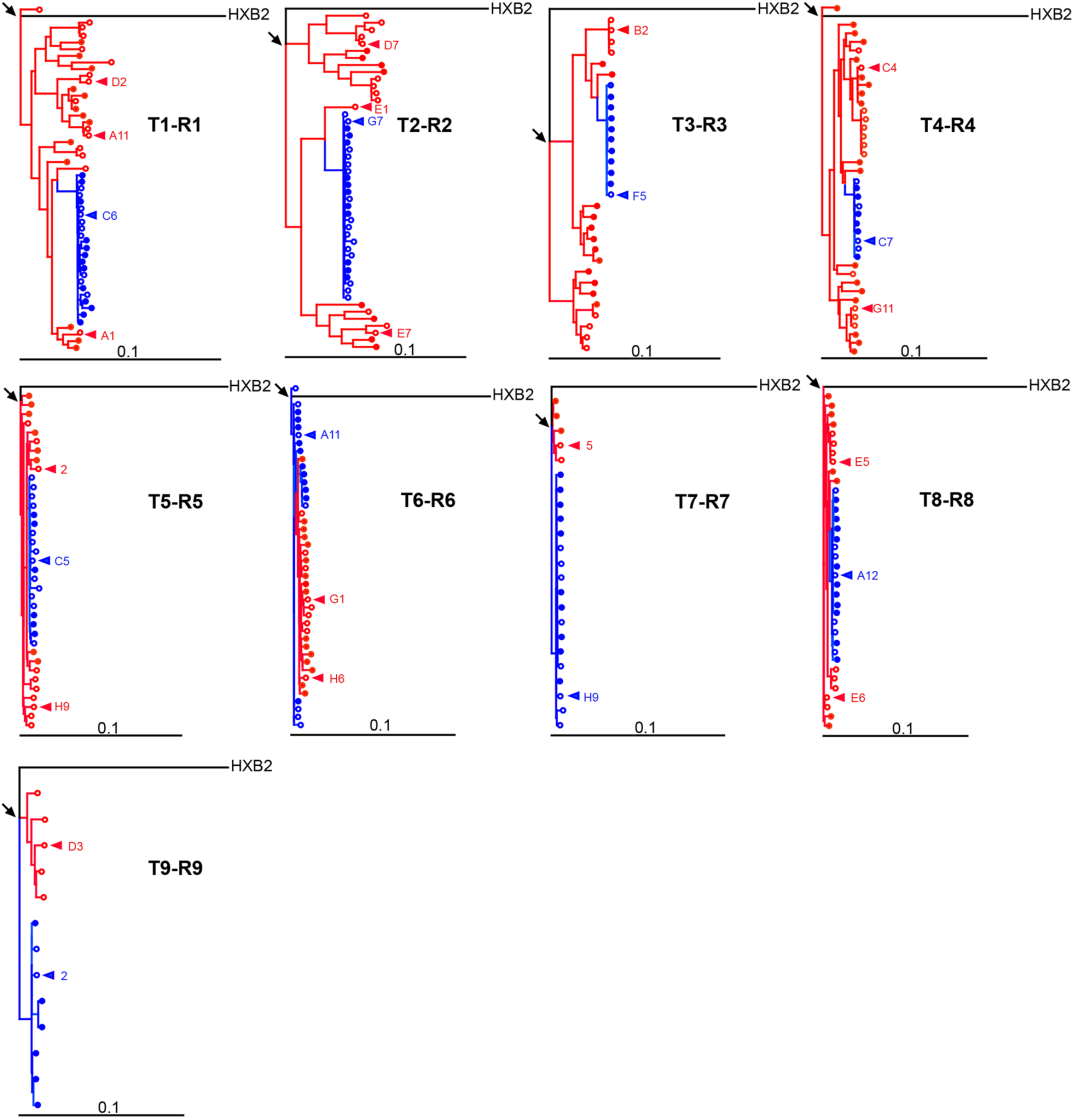
Linkage of *envelope* sequences from HIV-1 transmission pairs. Maximum likelihood phylogenetic trees of full-length *env* sequences from transmitters (*red*) and recipients (*blue*). Sequences derived from single genome amplification are displayed with *filled circles* and those inferred from cloning with *open circles*. *Triangles* depict sequences that were used as Env-pseudoviruses for follow-up experiments. *Arrows* indicate the most recent common ancestors (MRCA). Branch lengths are drawn to scale and HIV-1 HXB2 was used as a subtype B reference. For highlighter plots with all sequences see Additional file 4: Figure S4

To enable phenotypic comparison of the *env* gene of transmitter and recipient viruses we performed comparisons with virus isolates and Env pseudotyped viruses. All, the virus isolate, the cloned full length *envs* and the *env* SGA analysis were derived from the closest possible time points to the EDT. Virus isolates of recipients and transmitters were derived from peripheral blood mononuclear cells (PBMCs) by co-culture with CD8-depleted PBMCs from HIV-1 negative donors. Full length Env clones were isolated from plasma from the same time point the SGA analysis was performed and from virus isolates derived from exactly this time point (Additional file 2: Figure S2). Cloned *env* sequences tightly clustered with *env* sequences derived from SGA (Fig. 1) and with next generation sequencing data from plasma and virus isolates (Additional file 3: Figure S3). Recipient Env clones selected for further characterization closely matched the respective consensus SGA sequences. The Env clones were identical to the respective recipient SGA consensus sequence in three cases, had one amino acid mismatch in three cases, two amino acid mismatches in two cases and three amino acid mismatches in one case (Additional file 4: Figure S4). Of note, in two recipients the cloned Envs contained mismatches that were identical to a SGA variant and were found as a variant in corresponding transmitters. As expected in established infection, transmitter sequences had a considerable heterogeneity. Based on an extensive sequence and phylogenetic analysis we selected virus Env clones that reflect the diversity seen in transmitters and were also functional in supporting virus entry. The selected clones are indicated in Fig. 1 and Additional file 4: Figure S4.

In total, we included for each recipient a single Env clone and for transmitters one to three different Env clones in the subsequent Env-pseudovirus analysis (Fig. 1; Additional file 3: Figure S3; Additional file 4: Figure S4). All selected Env clones were functional as defined by Env-pseudovirus infection of TZM-bl cells. We next compared the infectivity of transmitter and recipient viruses by measuring the infectivity of serial dilutions of pseudovirus stocks on TZM-bl cells. Activity of the luciferase reporter measured as relative light units (RLU) per μl input of the respective virus stocks was recorded. The infectivity varied both amongst transmitters (101–4637 RLU/μl virus stock input) and recipients (46–5752 RLU/μl virus stock input) but was not statistically different across the two groups (p = 0.496; Wilcoxon matched-pairs signed rank test).

The comparison of next generation sequencing data from virus isolates and, if available, plasma virus to the SGA consensus of recipients revealed that the retrieved virus isolates and Env clones were indeed representative of the patients virus populations. In six recipients the haplotype was identical to the SGA consensus, in two recipients the haplotypes contained mismatches that were also found in SGA and clonal sequences and in one recipient with three haplotypes the major haplotype was identical to the SGA consensus. In transmitters the haplotypes found in virus isolates and plasma closely matched the SGA and clonal sequences showing that our virus isolates represent the circulating virus population in vivo.

To obtain a comprehensive picture of the virus populations of transmitters and recipients at the EDT we compiled all *env* sequences retrieved by SGA and cloning in a genetic analysis. Recipient sequences proved very homogeneous and formed a monophyletic sub-cluster within their corresponding transmitter sequences (for high diversity transmitters) highly suggesting that the recipients were infected by a single T/F virus in all nine pairs we investigated (Fig. 1; Additional file 3: Figure S3). In line with this, recipients had a significantly lower genetic diversity in *env* (median 0.18 %, range 0.07–0.35 %) compared to transmitters (median 1.08 %, range 0.34–4.61 %; p = 0.004; Table 2).

As length and glycosylation of Env variable loops have been implicated as selecting features in transmission [16–18, 22] we compared these domains across transmission pairs. Although differences in length and glycosylation were apparent in the V1V2 and V4 domains, there was no consistent trend in recipients towards reduction in length (p = 0.854 for V1V2 and p = 0.713 for V4) or glycosylation (p = 0.346 for V1V2 and p = 0.233 for V4; Additional file 5: Table S1). Two motifs, the presence of a Histidine at position 12 in the Env leader peptide associated with increased Env expression and infectivity, and the loss of a PNGS site in V4 (HXB2 position 413–415) whose presence is associated with neutralization escape, have previously been associated with acute infection [45, 46]. Additionally, acute viruses have been shown to bind with a higher affinity to the α4β7 integrin via a tripeptide motif in V2, most commonly Leucine-Aspartic acid-Isoleucine/Valine (HXB2 position 182–184) [32, 33, 47]. A comparison of transmitter and recipient Env sequences in our study did not link any of the described motifs with transmission as none of them were over- or underrepresented in recipient viruses (Additional file 5: Table S1).

Previously it was suggested that T/F viruses might harbor a more ancestral genotype as evidenced by shorter distances of T/F viruses to the most recent common ancestor (MRCA) [18, 48]. In six out of nine transmission pairs (T1–R1, T2–R2, T3–R3, T6–R6, T7–R7 and T9–R9) we found shorter distances of recipient *env* sequences to the MRCA as compared to transmitter sequences (Fig. 1, Additional file 6: Table S2). However, when performing a Wilcoxon matched-pairs signed rank test using the distances to the MRCA, we observed neither a statistical difference across all transmitter and recipient pairs (p = 0.129) nor when focusing on the pairs with high virus diversity (p = 0.188).

### Concurrent plasma antibodies of transmitters fail to neutralize transmitter and recipient viruses

**Fig. 2:**
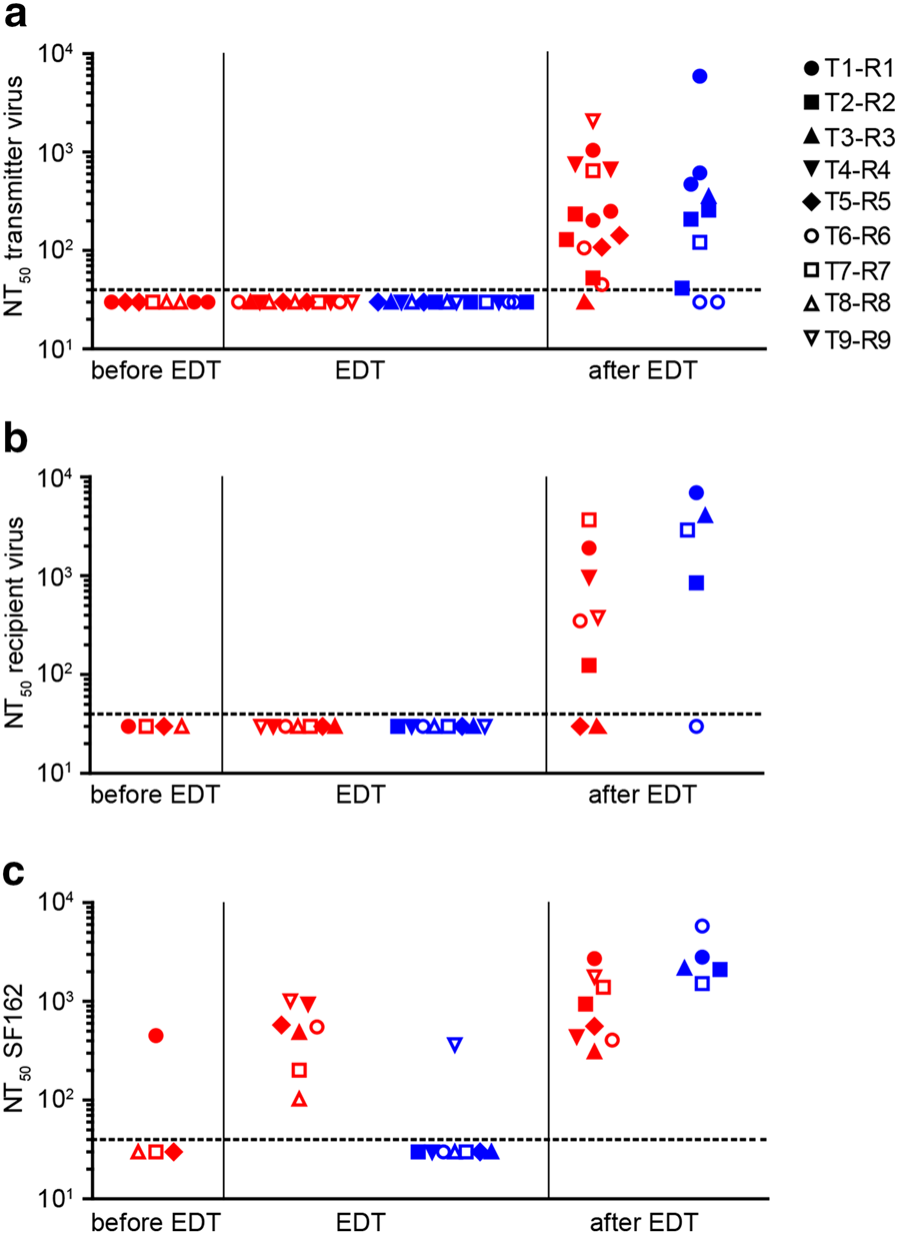
Transmitter and recipient viruses are resistant to plasma neutralizing antibodies of transmitters circulating at transmission. The 50 % neutralization titer (NT_50_, i.e., the reciprocal plasma dilution yielding 50 % neutralization) for **a** transmitter, **b** recipient and **c** SF162 Env-pseudoviruses against transmitter (*red*) and recipient (*blue*) plasma samples, respectively. Off-antiretroviral treatment (ART) plasma samples tested were from time points before the estimated date of transmission (EDT) (only for transmitter plasma), closest to the EDT or after the EDT. Due to differences in ART initiation and limitations in sample availability, plasma sample time points and virus combinations are not identical for each probed individual. NT_50_ values were derived from 2 independent experiments each performed in duplicates. SF162 was included as a Tier-1 neutralization sensitive virus. The *dotted line* indicates the threshold for NT_50_ determination as the highest plasma concentration tested was 1:40 and values below the threshold were given an arbitrary value of 30. For detailed NT_50_ values and for plasma sample time points see Additional file 8: Table S3 and Additional file 2: Figure S2, respectively

Sensitivity to neutralization has been considered a common property among transmitted virus variants [16]. To explore the neutralization sensitivity of transmitter and recipient Env-pseudoviruses, we examined the neutralization capacity of transmitter and recipient plasma in TZM-bl Env-pseudovirus assays. As expected, recipients’ plasma at the EDT displayed no noteworthy neutralization capacity as the plasma samples were all close to the EDT and an autologous neutralization response had not yet evolved (n = 8; Fig. 2a, b). Importantly, however, transmitter plasma from time points before the EDT (n = 4) and from the closest time point to the EDT (n = 7) neither neutralized the autologous nor recipient virus (Fig. 2a, b). At the highest plasma concentration tested, the seven probed transmitter plasma samples derived from the closest time point to the EDT showed only low level neutralization activity against the corresponding recipient and transmitter viruses in line with the expected neutralization escape in the transmitter. Of note, in five cases recipient viruses were slightly more neutralization sensitive than the corresponding transmitter viruses, however no consistent difference in neutralization sensitivity was observed between transmitter and recipient Envs (p = 0.297; Additional file 7: Figure S5).

To obtain an overall estimate of the neutralization capacity of the probed transmitter plasma samples we analyzed their activity against the Tier-1 HIV-1 isolate SF162 (Fig. 2c). Transmitter plasma samples from time points before the EDT neutralized SF162 only in one out of four cases recapitulating the early infection stage of individuals T5, T7 and T8. A basic neutralization response was established at later time points in all transmitters as all transmitter plasma samples from the EDT effectively neutralized SF162 (50 % neutralization titer (NT_50_) ranging from 103 to 1017; Fig. 2c). This indicates the presence of neutralizing antibodies in the transmitter plasma at the EDT to which the contemporaneous recipient virus is not sensitive (NT_50_ SF162 vs. recipient or transmitter virus p = 0.016). In contrast, recipient plasma samples, as expected, failed at the time point of transmission to neutralize SF162. The exception was patient R9 who neutralized SF162 with relatively high potency (NT_50_ = 363) indicating an early neutralizing antibody response in this individual (Fig. 2c). As expected most plasma samples of transmitters and recipients derived at time points after the EDT improved neutralization activity against the transmitter and recipient viruses derived at the EDT and also more potently neutralized SF162 (Fig. 2).

### Comparable sensitivity of transmitter and recipient viruses to neutralizing antibodies and entry inhibitors

**Fig. 3:**
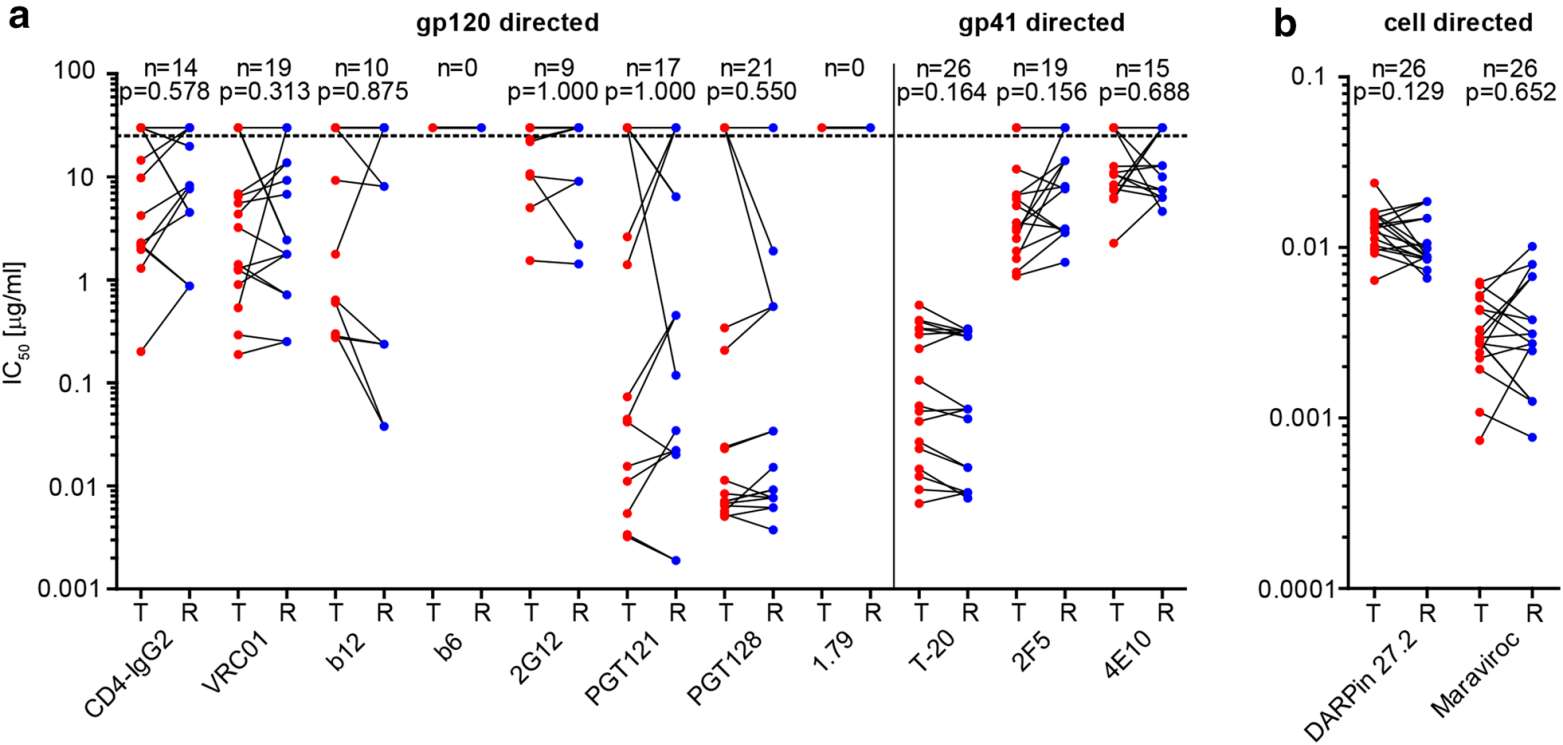
Transmitter and recipient viruses exhibit no difference in sensitivity to neutralizing antibodies and entry inhibitors. Inhibition of transmitter and recipient Env-pseudoviruses by **a** gp120 directed, gp41 directed and **b** cell directed inhibitors in TZM-bl assays. Transmitter (T) and recipient (R) 50 % inhibitory concentration (IC_50_) values in μg/ml are depicted and IC_50_ values above the highest concentration tested (indicated by the *dotted line*) were given an arbitrary value of 30. Transmission pairs are connected with a line. N specifies the number of Env-pseudoviruses (out of 26) with an IC_50_ value below the highest inhibitor concentration tested and the p values were determined by the Wilcoxon matched-pairs signed rank test. Values were derived from 2 independent experiments each performed in duplicates. For detailed IC_50_ values see Additional file 9: Table S4

Although sensitivity of recipient and transmitter viruses to the autologous and transmission partner plasma antibodies did not differ, this does not allow drawing general conclusions on the neutralization sensitivity of the respective strains, as this solely reflects that the probed viruses largely had escaped contemporaneous autologous neutralization activity. We thus performed a comprehensive assessment of the neutralization sensitivity of the recipient and transmitter viruses against a panel of neutralizing antibodies and entry inhibitors in TZM-bl Env-pseudovirus assays. We included in our inhibitor panel CD4 binding site directed agents (CD4-IgG2, VRC01, b12 and b6), the monoclonal antibody 2G12 that targets a carbohydrate motif in gp120, V3 directed antibodies (PGT121, PGT128 and 1.79) and three gp41 specific inhibitors (T-20, 2F5 and 4E10). In sum, transmitter and recipient viruses disclosed moderate sensitivity to the inhibitor panel tested (Fig. 3a, Additional file 9: Table S4). Furthermore, although differences in sensitivity between individual transmitter and recipient Env-pseudoviruses existed, we found no evidence for an overall pattern of neutralization sensitivity that was segregating recipient from transmitter viruses (Fig. 3a).

### Enhanced replicative capacity is not required for transmission

It has been considered for long that viruses that are successfully transmitted may have distinguishing properties such as an improved access to target cells in the genital tract (reviewed in [49]). We indirectly assessed the efficacy of the transmitter and recipient Env-pseudoviruses to infect cells at low CD4 and CCR5 levels by measuring their sensitivity to the CD4 specific inhibitor DARPin 27.2 and the CCR5 antagonist Maraviroc. Sensitivity to CD4 (p = 0.129) and CCR5 (p = 0.652) blocking was very similar across viruses from transmitters and recipients (Fig. 3b, Additional file 9: Table S4).

**Fig. 4:**
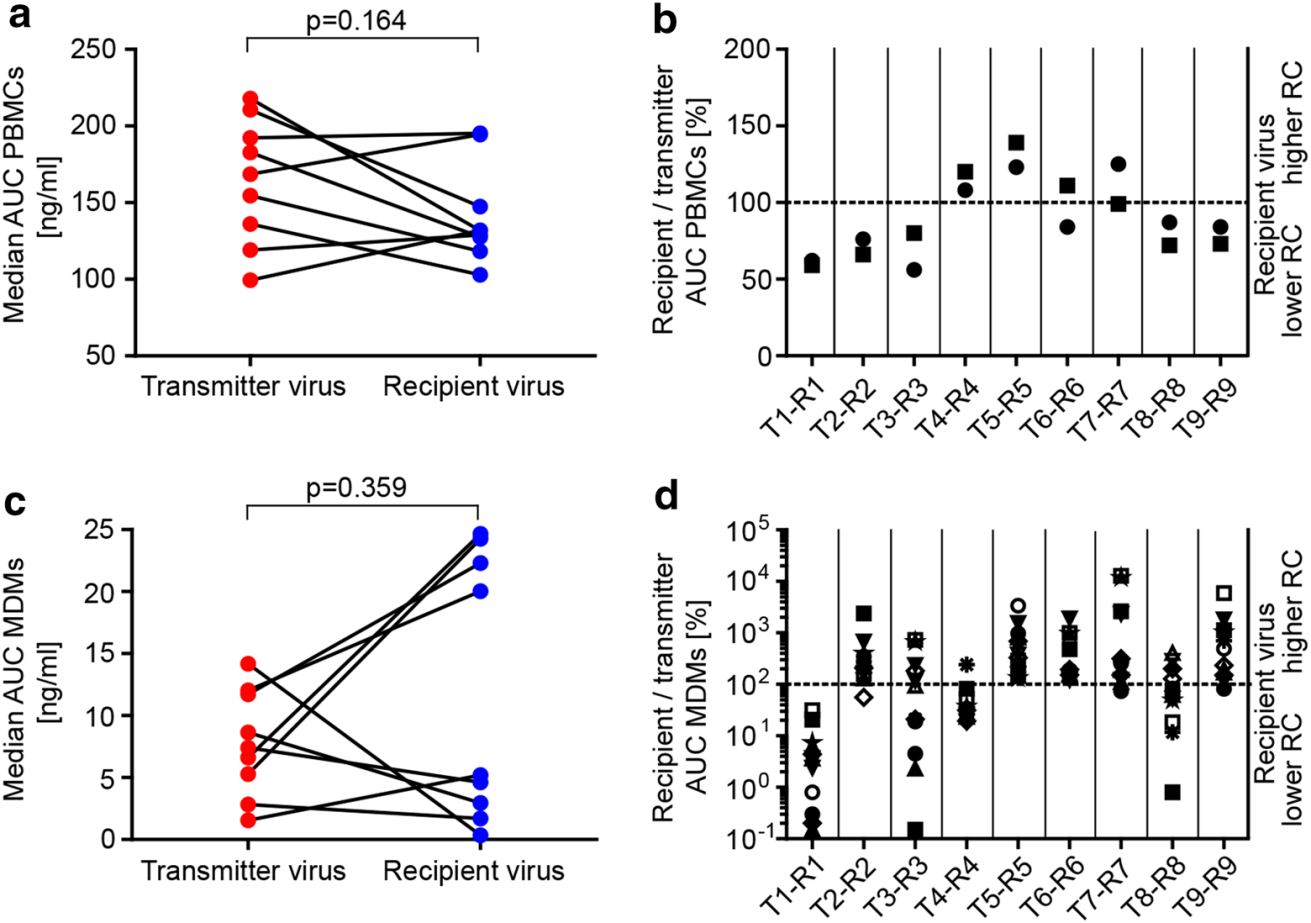
Transmitter and recipient viruses show no significant difference in replicative capacity in PBMCs and MDMs. Replicative capacity of transmitter and recipient virus isolates in CD8-depleted PBMCs and monocyte-derived macrophages (MDMs). Replicative capacity was quantified as area under the curve (AUC) of p24 antigen production in culture supernatants over a 14 day period. **a**, **c** Median AUCs for 2 PBMC pools and 12 MDM donors between transmitter and recipient viruses were tested for statistical significance with a Wilcoxon matched-pairs signed rank test. **b**, **d** AUCs of recipient virus isolates expressed relative to their corresponding transmitter virus isolate. Values above and below the *dotted line* indicate when recipient viruses have a higher and lower replicative capacity, respectively. Independent experiments are indicated by *different symbols*

To address target cell preference more directly, we next probed the infectivity of the transmitter and recipient virus isolates on primary CD4^+^ T-cells (PBMCs) and MDMs. A difference between these prototypic target cells of HIV-1 is the CD4 receptor density with CD4^+^ T-cells exhibiting approximately 20-fold higher CD4 densities than MDMs [50]. In order to limit the influence of donor variability in the readout, we probed the entire virus panel on two pools of stimulated PBMCs of three donors each and titrated each virus isolate in parallel in the same experiment. To derive a measure for the replicative capacity of individual strains we quantified virus replication by p24 antigen production and determined the area under the curve (AUC) over the entire 14 day observation period. A paired analysis of the derived median AUC revealed no significant differences between the transmitter and recipient viruses (p = 0.164; Fig. 4a). Alternative approaches to evaluate the replication fitness confirmed this (Additional file 10: Figure S6). To allow a comparison across transmission pairs we next calculated for each pair the ratio of recipient relative to transmitter AUC (Fig. 4b). These ratios showed a similar pattern with most recipients replicating at comparable levels relative to their transmitter in the two independent experiments. Transmission pairs T4–R4 and T5–R5 were the only two cases where the recipient viruses had a higher AUC on both donor pools (recipient AUC 8–39 % higher). In five transmission pairs, the recipient virus had a lower AUC than the corresponding transmitter (recipient AUC 13–44 % lower).

To probe the capacity of the viruses isolated from the transmission pairs to infect MDMs we compared their replicative capacity on MDM preparations from twelve different donors as susceptibility to infection is known to vary substantially. In sum, we noted no general pattern for preferential replication in MDMs by transmitter or recipient virus isolates (p = 0.359; Fig. 4c). Despite the high donor variability of MDMs we observed a congruent pattern: In five pairs recipient viruses proved to have a higher replicative capacity on MDMs and in four pairs the transmitter virus showed equal (T3–R3 and T8–R8) or higher (T1–R1 and T4–R4) infection of MDMs (Fig. 4d). High replicative capacity on PBMCs and MDMs was not always linked. Transmitter T1 as well as recipient R5 had higher activity than their transmission partners both on PBMCs and MDMs, whereas for other pairs higher infectivity on one cell type was associated with a lower infectivity on the other (T2–R2, T4–R4 and T9–R9).

### Entry kinetics of transmitter and recipient viruses match closely

**Fig. 5:**
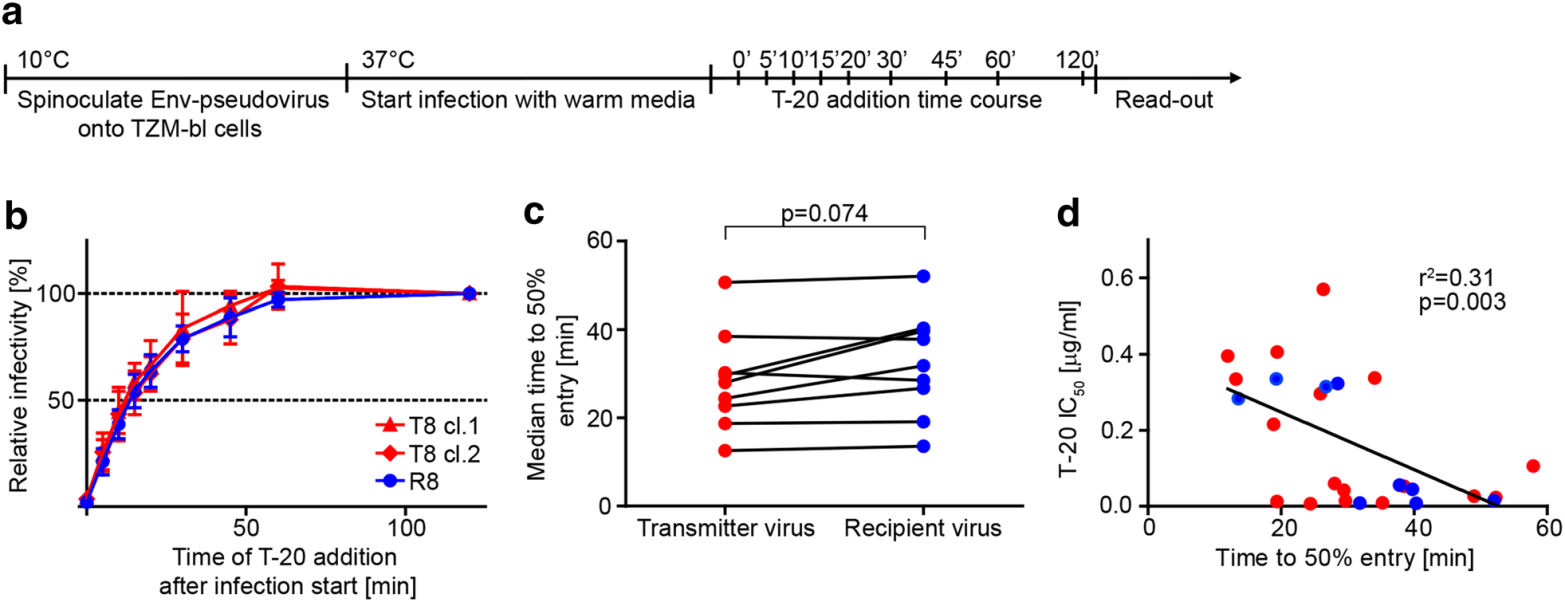
Transmitter and recipient viruses show comparable entry kinetics. **a** Scheme of time-of-inhibitor addition experiment on TZM-bl cells. **b** Entry kinetic curves for one representative transmission pair with two transmitter Env-pseudoviruses depicted in *red* and the recipient Env-pseudovirus in *blue*. The relative infectivity reached after 120 min was set as 100 % and data points were normalized to this value. Data points are mean and standard deviation from three independent experiments each performed in duplicates. **c** Time to 50 % entry was determined out of entry kinetic curves and median time to 50 % entry of transmitter Env-pseudoviruses was compared to recipient Env-pseudoviruses applying a Wilcoxon matched-pairs signed rank test. **d** Linear regression analysis of T-20 IC_50_ and time to 50 % entry with r^2^ and p value depicted. Data for transmitters and recipients are indicated in *red* and *blue*, respectively

A potential step where selection of recipient viruses may occur is the entry process. Viruses that are capable of completing entry more efficiently may have an advantage in establishing infection. To probe this, we assessed the entry kinetics of transmitter and recipient Env-pseudoviruses using a time-of-inhibitor addition experiment on TZM-bl cells [51]. In this assay, infection is synchronized through spinoculation and initial infection arrest at 10 °C and therefore allows for the assessment of virus entry kinetics at the steps post-attachment but not during attachment. Infection is initiated upon temperature increase to 37 °C and blocked at different time points by addition of the fusion inhibitor T-20 (Fig. 5a). By normalizing data points to the relative infectivity reached after 120 min and plotting entry kinetic curves (Fig. 5b), the time to attain 50 % entry was determined for each Env-pseudovirus tested. Time to 50 % entry ranged for transmitters from 12 to 58 min and for recipients from 14 to 52 min (Additional file 11: Figure S7), and the medians were similar between both groups (Fig. 5c). We observed a significant negative association between the time to 50 % entry and the T-20 50 % inhibitory concentration (IC_50_; r^2^ = 0.31, p = 0.003), indicating that the fusion inhibitor benefits from slow entry kinetics likely as this widens its window of action (Fig. 5d).

### Transmitter and recipient viruses are not distinguished by their cell–cell transmission and free virus infection capacity

**Fig. 6:**
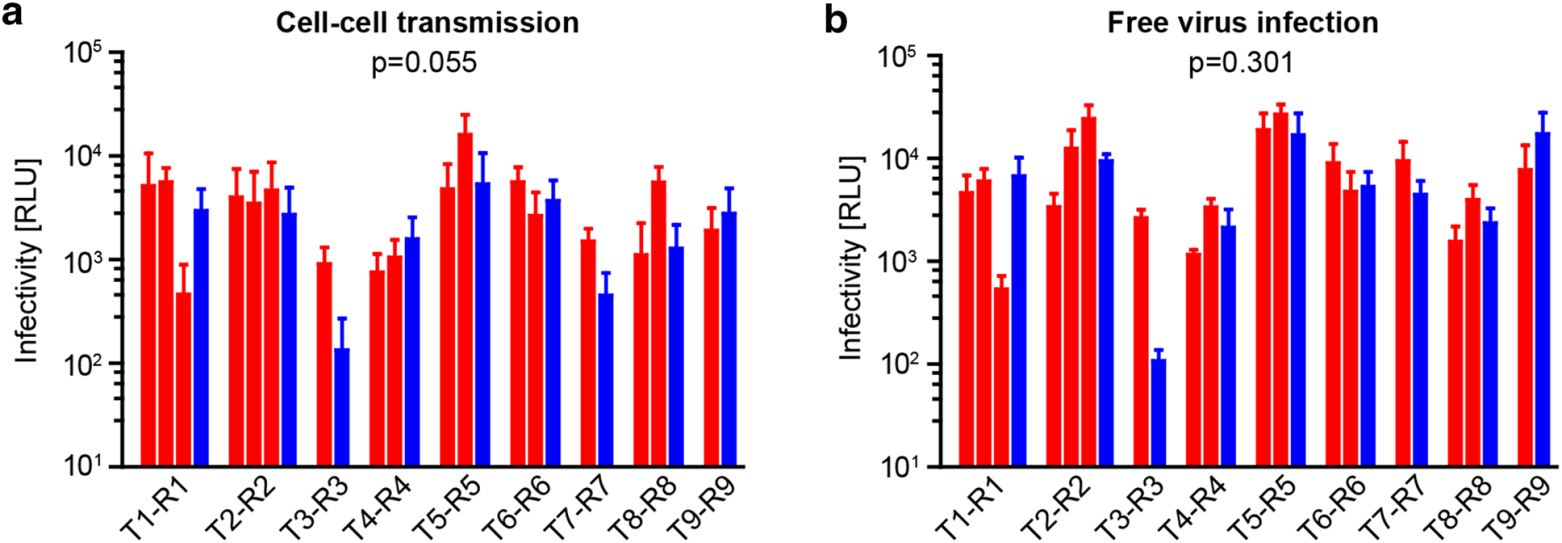
Transmitter and recipient viruses have comparable cell–cell transmission and free virus infection capacities. **a** Cell–cell transmission and **b** free virus infection of transmitter (*red*) and recipient (*blue*) Env-pseudoviruses are shown. For cell–cell transmission pseudovirus transfected 293-T cells (gaussia luciferase reporter NLinGluc) and for free virus infection 293-T derived pseudovirus (firefly luciferase reporter NLluc-AM) were used. A3.01-CCR5 served as target cells. Mean and standard deviation from three to six experiments each performed in triplicates are shown. Statistical significance between transmitter virus median values and recipient viruses was tested with a Wilcoxon matched-pairs signed rank test

A further phenotype that might endow recipient viruses with a transmission benefit is a higher efficacy for HIV-1 cell–cell transmission and/or free virus infection. To compare both entry modes between transmitter and recipient Env-pseudoviruses, we used 293-T cells as donor cells and assessed cell–cell and free virus infection of A3.01-CCR5 target cells as in previously described protocols [52, 53]. Our data showed that in most transmission pairs the pattern of infectivity in cell–cell and free virus transmission was concordant. Furthermore, recipient and transmitter viruses displayed variable cell–cell and free virus infection capacities. When we compared median values of transmitters to values of recipient viruses, neither cell–cell transmission (p = 0.055; Fig. 6a) nor free virus infection capacity (p = 0.301; Fig. 6b) was consistently different between transmitter and recipient viruses.

### Recipient viruses are more sensitive to IFNα

Resistance to IFNα induced antiviral defenses has been postulated to steer the foundation of recipient viruses [34, 35]. Two other studies however, could not reproduce higher IFNα resistance of acute viruses [26, 36].

**Fig. 7:**
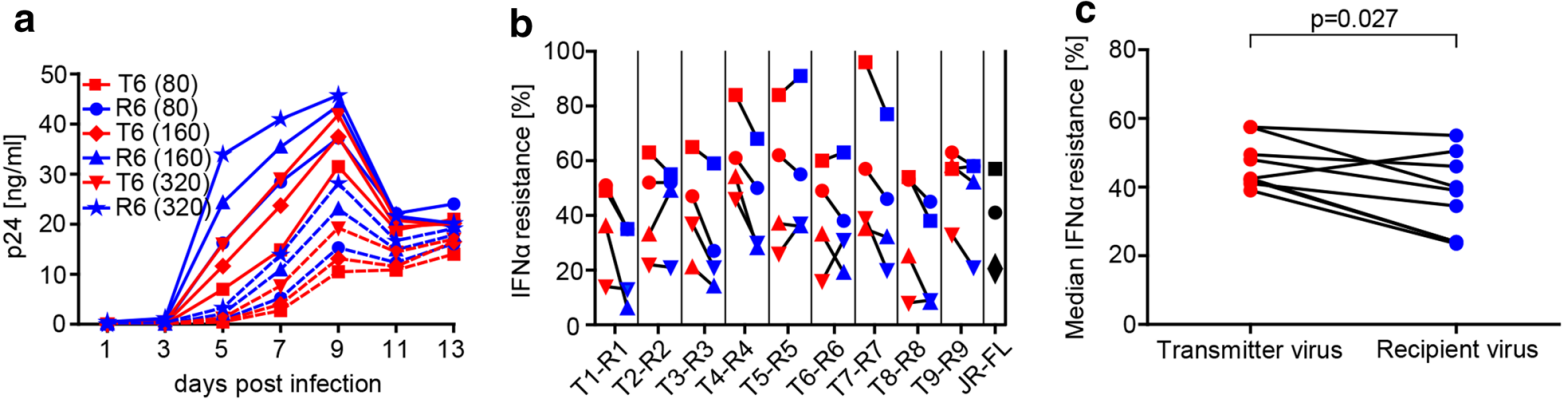
Recipient viruses exhibit a higher sensitivity to IFNα. **a** Profile of p24 antigen production measured in culture supernatants of CD8-depleted PBMCs infected with virus isolates of transmission pair T6–R6 at three different input TCID_50_’s of 80, 160 or 320. The *solid line* represents replication in absence and the *dashed line* replication in presence of 1000 U/ml IFNα, respectively. **b** IFNα resistance defined as area under the curve (AUC) of p24 antigen production over a 13 day period for transmitter and recipient virus isolates on PBMCs in the presence of 1000 U/ml IFNα expressed as % of AUC in its absence. Four independent experiments indicated by different symbols are depicted. **c** Median IFNα resistance of four experiments between transmitter and recipient viruses was probed with a Wilcoxon matched-pairs signed rank test. Data for transmitters and recipients are indicated in *red* and *blue*, respectively

To clarify if IFNα resistance is a property that provides selective advantage during transmission, we analyzed virus isolates from transmitters and recipients for their sensitivity to IFNα in in vitro infection experiments of PBMCs. To account for the high variability in PBMC infectivity, we determined the 50 % tissue culture infectious dose (TCID_50_) of all virus isolates on the same donor PBMCs and performed the experiment on four different PBMC pools. Assessment of the influence of type I IFN on HIV-1 infectivity necessitates a tightly controlled system as the effect of IFN is relatively modest and even small differences in virus input can result in substantially different accuracies in the estimation of IFNα activity as shown for the transmission pair T6-R6 (Fig. 7a). In line with this we observed a strong association between replicative capacity in the absence and in the presence of IFNα (Additional file 12: Figure S8). We estimated the AUC over the entire observation period and determined the IFNα resistance defined as the percentage of replication (AUC) in the presence of IFNα compared to the untreated control. While we observed variation due to donor cell variability (Fig. 7b) the overall picture proved very coherent. In contrast to previous observations [34, 35] the median IFNα sensitivity proved to be higher for recipient viruses than their paired transmitter viruses in our cohort (p = 0.027) (Fig. 7c), suggesting that resistance to IFNα was not decisive for the establishment of recipient viruses. Of note, across the nine transmission pairs investigated, we found no association between the sensitivity to IFNα mediated restrictions and the replicative capacity of virus isolates (Additional file 12: Figure S8).

### Influence of low and high diversity transmission

Low diversity transmitters are expected to harbor viruses that are phenotypically more closely related to the recipient viruses than transmitter viruses with high diversity from chronic infection. In our study four transmitters with low virus diversity (*env* diversity < 1 %) were included (T5, T6, T7 and T8). Due to their high sequence similarity with the recipient these cases are intriguing to study as they may allow to pinpoint domains that are under specific selection more accurately. On reverse, if adaptation in the transmitters was too short to allow adaptation to the new host, these acute transmissions may indeed be solely stochastic events and selection only evident in chronic transmission. To verify the impact of *env* diversity in the transmitters we analyzed all data sets for all nine transmission pairs combined and in a separate analysis focusing only on either high or low diversity transmission pairs using different thresholds (Additional file 13: Table S5). In line with what we observed for the combined nine pairs, we detected no consistent phenotypic difference of recipient viruses when focusing exclusively on the high diversity transmission pairs or on the low diversity transmission cases (Additional file 13: Table S5).

## Discussion

Discoveries of recent years established that commonly only a single viral variant out of a genetically diverse virus population in the transmitting partner establishes HIV-1 infection in the new host [11–15, 39]. It is evident from this that HIV-1 faces a strong population bottleneck upon transmission and available data suggest that this is most stringent during heterosexual female-to-male transmission [48]. To what extent the transmission process selects for viruses with specific properties or is simply the result of a stochastic process has not been fully resolved (reviewed in [6, 7]). Several viral features have been implicated with transmission success, such as CCR5 tropism [1, 8–11, 20], a loss of glycosylation sites and a reduction in length of variable loops [16–18, 22, 23], a higher resistance to IFNα [34, 35] and increased Env incorporation [34]. However, to date no consistent picture has emerged if and which of these viral properties are decisive in determining the selection of viral variants during mucosal transmission.

Delineating HIV-1 transmission is inherently difficult as the earliest events of transmission cannot be captured and only a limited number of confirmed transmission pairs have been identified. Much of the current knowledge is thus based on cross-sectional studies of T/F viruses in comparison with unrelated chronic control viruses. Nevertheless, these cross-sectional studies defined virus properties associated with transmission despite the high genetic diversity of unrelated virus strains. In linked transmission pairs differential properties should therefore be even more pronounced and potentially easier to define which prompted us to perform the current study. Transmitter and recipient viruses in our study represented the respective circulating virus as determined by SGA analysis and the viruses from the transmission pairs were closely related based on both *env* and *pol* sequence analyses. All transmission pairs were confirmed based on clinical data, patient demographics, self-reporting of exposure and EDT determined based on laboratory data. Our paired analysis of transmitters and recipients included nine subtype B MSM and MTF transmission pairs. Recipient viruses proofed resistant to plasma antibodies circulating in the corresponding transmitter at the time of transmission underlining that a neutralization escape variant was transmitted. Our data support findings in subtype B MSM [19] and subtype A, C and D mother-to-child transmission [54–56], where no selection for neutralization sensitive viruses occurred. Although transmission of neutralization sensitive viruses may be possible as shown for heterosexual subtype C transmission pairs [16, 26], our data support the notion that selection for neutralization sensitive variants cannot be a general determinant of mucosal HIV-1 transmission.

Since the transmission partners’ immune responses are subject to viral escape, resistance to the autologous response does not allow conclusions on the general neutralization sensitivity of the virus strains. Previous reports in the literature remained inconclusive on whether T/F viruses have distinct neutralization properties [11, 20, 24, 25]. Our comprehensive neutralization sensitivity tests with a panel of gp120, gp41 and cell directed antibodies and inhibitors revealed, however, no difference in neutralization sensitivity of recipient and transmitter viruses.

Detailed sequence comparison of the *env* gene of transmitters and recipients also uncovered no consistent differences in variable loop length and glycosylation. As expected, transmitter sequences were more heterogeneous with regard to length and PNGS in the V1V2 and V4 regions, particularly in chronic transmitters. There was, however, no prioritization amongst recipients for shorter variable loops or lower glycosylation in accordance with previous findings for subtype B transmission [17, 19–21].

Furthermore, we could not identify a difference in the conservation of the tripeptide motif responsible for α4β7 integrin binding between recipients and transmitters. This does however not exclude that differences in exposure of the tripeptide motif may exist that only functional assays that assess α4β7 integrin binding or replication in α4β7 expressing cells may reveal [32, 57].

In six out of nine transmission pairs recipient Envs showed shorter distances to the MRCA when compared to transmitters as previously suggested [18, 48], yet this difference was not statistically significant. Supporting this, across all phenotypic and genotypic parameters we assessed recipient viruses closer to the MRCA were indistinguishable from transmitters (Additional file 13: Table S5).

A detailed analysis of the entry fitness of the transmitter and recipient viruses further highlighted that there is no general trend towards selection of viruses with specific entry properties. In the cases studied, transmission did not favor viruses with faster entry kinetics which is in agreement with a previous cross-sectional study of HIV-1 subtype B viruses [20]. Furthermore, recipient viruses were also indistinguishable from transmitter viruses in their cell–cell transmission or free virus infection capacities. Likewise we observed no general trend towards selection of viruses with higher replicative capacity on primary cells (PBMCs and MDMs) again supporting earlier findings [20, 24, 26, 28]. In contrast, a recent study by Carlson and colleagues found in a sequence based modelling study of 137 linked heterosexual transmission pairs that transmission of more consensus-like variants predicted increased in vivo fitness in the new host [48].

While most of the features we studied focused solely on the impact of the *env* gene in transmission, the replicative capacity of primary isolates will also be influenced by other factors including the efficacy of the reverse transcription and integration process or the virus’ capacity to counteract intrinsic defense mechanisms of the host cell. The latter has gained particular attention in the transmission setting following reports that suggested that recipient viruses are more resistant to the action of IFNα [34, 35]. Improved evasion of the intrinsic immune defense certainly can be envisaged to provide a selection advantage. When we probed the sensitivity to IFNα across our transmission pairs, we observed however no such trend. On the contrary, while sensitivities on the individual level differed, across all patients recipient viruses proved more sensitive to IFNα than transmitter viruses. As IFNα mediated control of HIV-1 is not very potent and influenced by subtle differences in virus input and replicative capacity, we chose assay conditions that ensure that our data on IFNα sensitivity is not confounded by differences in replication. It is important to note that earlier studies which observed a higher resistance of recipient viruses to IFNα, did not have the possibility to investigate matched transmission pairs which could in part influence conclusions as individual sensitivities can vary substantially [34, 35]. Supporting our finding, a recent publication investigating six HIV-1 subtype C transmission pairs also observed no increased resistance of T/F viruses [26]. Furthermore, Etemad and colleagues found in injection drug users of HIV-1 subtype B an increased IFNα sensitivity of acute Envs as compared to chronic Envs [36].

The differences in IFNα sensitivity we observed between transmitters and recipients were not pronounced. We thus interpret the fact that recipient viruses were more sensitive to IFNα not as a result of positive selection of these viruses but rather as an indication that the IFN response may not play a critical role during the earliest stages of infection leading to the establishment of the T/F viruses. That notwithstanding, IFN responses certainly play a crucial role in established infection as evidenced by the increasing number of antiviral factors that are regulated by the type I IFN response (reviewed in [58]). In vivo transcriptome studies have shown increased IFN expression in patients with high viral loads [59] proposing that escape from IFN triggered restriction factors may occur during the course of infection as suggested in HIV/Hepatitis C virus co-infected patients following treatment with pegylated IFNα/ribavirin [60, 61]. Due to the persistent exposure of viruses to IFN, resistant viral variants may evolve and the virus population in transmitters likely reflects a very heterogeneous population in this respect. Depending on which viral gene is affected by the escape to restriction factors this may also impact on the viral fitness and provide a selection disadvantage in an environment where IFN activation has not yet occurred.

We used low-passage virus isolates to study phenotypic properties over multiple rounds of replication, namely infection of PBMCs and MDMs as well as sensitivity to IFNα during infection of PBMCs. While the use of virus isolates does not allow the focus on a specific gene or virus variant, as the fidelity of all virus genes will impact infectivity and a range of variants will be present in a given stock, this variety also provides opportunities for analysis. In addition virus isolates provide insight on the replication competent virus population circulating in a patient at a given time point. As a main interest of our study was on the *env* gene, we sequenced *env* genes of all virus isolates and found them to closely match the defined clonal variants of the respective patients (Additional file 4: Figure S4) further validating the use of these isolates in infectivity experiments. Full-length genome constructs as compared to virus isolates may be a useful tool to closely associate genotypes to phenotypes and to detect subtle differences more precisely. However, selecting IMCs that represent the diversity present in the transmitter remains challenging.

We identified five transmission pairs where transmitters harbored a high virus diversity (env diversity > 1 %) and four with low diversity (<1 %) that occurred in transmission during acute phase or early after drug cessation. As the latter scenarios are a main source of virus transmission [42–44], we thought it prudent to include the low diversity pairs in our analysis. Analyzing both high and low diversity transmission pairs may open advanced possibilities to trace potential bottlenecks. While in general selection may be easier to define in high diversity pairs, defining the important genotypic changes associated with the phenotypic profiles may benefit from analysis in the low diversity setting. We thus performed two separate analyses, one for all pairs grouped together and one for only high diversity transmission pairs. Similar to the observation with all nine transmission pairs, we found amongst high diversity pairs no phenotypic property that segregated transmitter from recipient viruses. As with most other studies in the field, the number of transmission pairs identified was small reducing statistical power to make definite conclusions. Yet, the fact that we saw no strong patterns suggests that if during transmission selective forces are present, they are likely to be subtle hence will require larger cohorts of transmission pairs than currently available to further unravel selective properties during transmission.

In sum, with the possible exception of increased sensitivity to IFNα, our study revealed no phenotypic feature that was linked with transmission strongly suggesting that transmission is to a large proportion stochastic. Nevertheless, this does not rule out that selective determinants exist. We consider it thus highly important that larger studies are conducted to further explore potential factors involved in transmission. These factors may vary in type and extent of influence depending on the HIV-1 subtype, mode of transmission and infection status of the transmitter and hence large cohorts of confirmed linked transmission pairs that cover all these settings need to be established and their viral properties explored. Our study further highlights the complexity of the T/F analysis and the need for meta studies incorporating published transmission pairs to identify T/F reference variants and key methods to build on and to eventually clarify the influence of viral properties in transmission.

## Conclusions

HIV-1 transmission is characterized by a constraint in the number of viral variants establishing infection. Several viral properties including CCR5 tropism, a reduction in length and glycosylation of Env variable loops, replicative capacity and resistance to IFNα have been associated with selection at the transmission bottleneck. However, if and which of these many phenotypic properties truly govern transmission has not been conclusively resolved as the investigated cohorts often lacked information on linked transmission partners, or cohorts differed in the transmission mode and the subtype studied. To investigate selective forces of transmission, we identified nine HIV-1 subtype B MSM and MTF transmission pairs and assessed their genotypic and phenotypic properties of transmitter and recipient viruses isolated at closest possible time points to transmission.

The viral features we investigated include besides an extensive sequence analysis neutralization sensitivity, replicative capacity in different cell types and sensitivity to IFNα. Despite this wide repertoire we found with the exception of increased IFNα sensitivity, that none of the other properties consistently distinguished recipient from transmitter HIV-1 subtype B viruses suggesting that transmission is to a considerable extent stochastic. Defining and fine-tuning viral factors that govern transmission will thus remain challenging and warrants further investigations in larger cohorts of confirmed transmission pairs.

## Methods

### Patients

Patients were enrolled in the Zurich Primary HIV Infection study (ZPHI) and/or the Swiss HIV Cohort Study (SHCS). The ZPHI is an observational, non-randomized, single center cohort that includes patients with a confirmed acute or recent primary HIV-1 infection (www.clinicaltrials.gov, ID NCT00537966) [37–39]. Patient visits, blood collections and sampling of plasma and PBMCs are scheduled every 3 months. The SHCS is a nationwide, clinic-based cohort with semiannual visits and blood collections enrolling all HIV-infected adults living in Switzerland [40]. The SHCS is estimated to include at least 53 % of all HIV infections ever diagnosed in Switzerland of whom 79 % were screened for drug resistance since 1996 [40, 41]. The ZPHI study and the SHCS have extensive biobanks. The estimated date of transmission (EDT) was established for every transmission pair by the inclusion of clinical and laboratory data from ZPHI patients such as known risk situations, appearance of first symptoms, earlier negative test results, avidity assays and Western blot results as described in detail elsewhere [37, 38]. Acute and recent infections were defined as a documented seroconversion within 90 and 180 days after the EDT, respectively [37]. Chronic infections were defined as a minimum of 180 days since seroconversion. Patient characteristics are listed in Table 1. Three transmission pairs could be verified clinically because the infected partners accompanied the acutely infected patient to the clinic and disclosed the transmission history (T5–R5, T8–R8 and T9–R9).

### Phylogenetic and sequence analysis

Phylogenetic linkage was assessed between individuals enrolled in the ZPHI and the SHCS using all available *pol* gene sequences derived from genotypic drug resistance tests (years 1995–2012) from the Swiss HIV drug resistance database. Population-based sequencing of the *protease* and the *reverse transcriptase* is done by four laboratories authorized by the Federal Office of Public Health as described previously [62]. We used R software environment for statistical computing (http://www.R-project.org), HMMalign [63], PHYLIP 3.68 (distributed by J. Felsenstein, University of Washington, Seattle) and a collection of Unix shell scripts and Perl scripts. Subsequently, therapy history, viral load time courses and the EDT were used to verify a potential transmission cluster. From these derived putative transmission pairs, confirmatory single genome amplification and sequencing of full-length *env* and phylogenetic analysis thereof was done. Pairwise genetic distances and viral diversities were calculated by MEGA5 using the Tamura-Nei model [64]. Neighbor joining phylogenetic trees were constructed with MEGA5 using the bootstrap method (1000 replications). Maximum likelihood phylogenetic trees were inferred with Dnaml (PHYLIP 3.68) using randomized input order, global rearrangements and multiple jumble options. For construction of phylogenetic trees following HIV-1 subtype reference sequences were used: B [GenBank:K03455], A1 [GenBank:AF004885], D [GenBank:K03454], C [GenBank:U52953] and F1 [GenBank: AF077336]. V1V2 and V4 domains were assigned for each sequence relative to HXB2 (HIV sequence compendium 2014). PNGS in V1V2 and V4 were determined using Glycosite (www.hiv.lanl.gov). Highlighter plots were generated with Highlighter for Amino Acids v1.3.4 (www.hiv.lanl.gov). The between group mean distance of transmitter and recipient *env* sequences to the MRCA sequence was calculated in MEGA5 using the Tamura-Nei model.

### Single genome amplification and sequencing

Single genome amplification and sequencing were performed according to previously described protocols [65]. Briefly, viral RNA was extracted from plasma using the RNeasy Kit (Qiagen) and reverse transcribed to cDNA using Superscript III (Life Technologies) and primer MascN (5′-CTGCCAATCAGGGAAGTAGCCTTGTGT-3′). To get an approximation of the quantity, cDNA was subjected to real-time PCR analysis. Subsequently, the cDNA was diluted to the endpoint so that no more than 30 % of the wells were PCR product positive. Two sequential PCR amplification reactions covering full-length *env* (nucleotides 6183–9096 based on HIV-1 HXB2) were then performed with Platinum Taq High Fidelity Polymerase (Life Technologies) according to the manufacturer’s instructions. Outer primers were MascA (5′-CACCGGCTTAGGCATCTCCTATGGCAGGAAGAA-3′) and MascN. The first PCR product was diluted 10 times for the second PCR. Primers for the nested second PCR were Ri29 (5′-GGTTAATTGATAGACTAATAGAAAGAGCAG-3′) and MascM (5′-TAGCCCTTCCAGTCCCCCCTTTTCTTTTA-3′). The PCR cycle conditions for both amplifications were: 94 °C for 2 min followed by 35 cycles of 94 °C for 15 s, 55 °C for 30 s, and 68 °C for 4 min, followed by a final extension at 68 °C for 10 min. PCR amplicons were gel purified with the QIAquick gel extraction kit (Qiagen) before sequencing. Full-length single genome sequences were derived by merging bidirectional sequences of 10 overlapping regions generated by dye terminator cycle sequencing (ABI Prism BigDye, Applied Biosystems). Sequences were edited with SeqMan (DNASTAR Inc., Wisconsin, USA) and checked for reading errors, premature stop codons and hypermutations with Hypermut (www.hiv.lanl.gov).

### Cells

293-T cells were obtained from the American Type Culture Collection (ATCC). TZM-bl and MT-2 cells were obtained from the NIH AIDS Reagent Program (NIH ARP). A3.01-CCR5 cells were described previously [66]. 293-T and TZM-bl cell lines were cultivated in DMEM containing 10 % heat-inactivated FCS and 1 % penicillin/streptomycin (P/S). MT-2 and A3.01-CCR5 cells were cultivated in RPMI 1640 containing 10 % heat-inactivated FCS and 1 % P/S. For TZM-bl infection experiments cell culture medium was supplemented with 10 μg/ml diethylaminoethyl-dextran (DEAE-dextran).

Stimulated primary CD8-depleted PBMCs were prepared as described [67]. Briefly, buffy coats from three healthy blood donors were CD8^+^ T cell depleted and PBMCs were isolated. Cells were adjusted to 4 × 10^6^ per ml with RPMI 1640 containing 10 % heat-inactivated FCS, 10 U/ml IL-2 and 1 % P/S, divided into three portions and stimulated with either 5 μg/ml phytohemagglutinin, 0.5 μg/ml phytohemagglutinin or OKT3. After 48 h, cells from all three stimulations were pooled and cultivated in RPMI 1640 containing 10 % heat-inactivated FCS, 50 U/ml IL-2 and 1 % P/S for further experiments.

To generate MDMs, CD14^+^ monocytes were isolated from CD8^+^ T-cell depleted PBMCs using CD14-coated magnetic microbeads (Miltenyi Biotec). Purity was checked by flow cytometry and monocytes were cultured in a 96-well plate at a density of 7 × 10^4^ per well in RPMI 1640 medium containing 10 % human serum (Sigma), 1 % P/S and 20 ng/ml macrophage colony-stimulating factor (M-CSF) (Peprotech). After 6 days, media was changed to RPMI 1640 containing 5 % human serum and 1 % P/S.

### Reagents

We thank following individuals for kindly providing antibodies or antibody expression plasmids: D. Burton, The Scripps Research Institute, La Jolla, USA for b12 [68], b6 [69], PGT121 and PGT128 [70]; D. Katinger, Polymun Scientific, Vienna, Austria for 2F5 [71], 4E10 [72, 73] and 2G12 [74]; J. Mascola, NIH, Bethesda, USA for VRC01 [75]; M. Nussenzweig, the Rockefeller University, New York, USA for 1.79 [76]. Antibody was produced by expression in 293-F cells and purified by protein G affinity and size exclusion chromatography [77]. DARPin 27.2 was produced as described [78]. CD4-IgG2 was provided by Progenics Pharmaceuticals. T-20 and Maraviroc were purchased from Roche Pharmaceuticals and Pfizer, respectively.

### Viruses

Autologous virus was isolated by co-culturing patient CD4^+^ T cells with stimulated, CD8-depleted PBMCs. The TCID_50_ on PBMCs and the co-receptor usage on MT-2 cells of the obtained virus isolates were determined as described previously [79].

For the generation of Env-pseudoviruses, viral RNA from plasma or virus isolates was isolated using the Rneasy kit (Qiagen) and reverse transcribed into cDNA using Superscript III (Life Technologies) and primer MascN. Subsequently, full-length *env* (nucleotides 5978–9171 based on HXB2) was PCR amplified with Platinum Taq High Fidelity Polymerase (Life Technologies) according to the manufacturer’s instructions. Primers for amplification were 5′allspl (5′-AAGAAGCGGAGACAGCGACGAAGA-3′) and MascN. The PCR cycle conditions were: 94 °C for 2 min followed by 35 cycles of 94 °C for 30 s, 58 °C for 30 s, and 68 °C for 3.5 min, followed by a final extension at 68 °C for 10 min. PCR products were gel purified and cloned into the pcDNA3.1/V5-His-TOPO expression vector (Life Technologies). Transformation into *E. coli* XL10 Gold (Agilent) was done according to the manufacturer’s instructions. Control of plasmid insert orientation via *env* PCR, plasmid preparation, Env-pseudovirus generation in small scale for functionality test and subsequent functionality screen on TZM-bl cells were performed as described [80]. Furthermore, clones were sequenced by Illumina MiSeq. For large scale Env-pseudovirus production, T75 cell culture flasks with 293-T cells were co-transfected with an HIV-1 backbone plasmid carrying the luciferase reporter gene (pNLluc-AM, [77]) and the respective *env* plasmid at a ratio of 3:1 using polyethylenimine (PEI) as described [81]. 48 h after transfection, virus supernatant was harvested and filtered. The infectivity of serial dilutions of pseudovirus stocks expressing the different Envs was determined on TZM-bl cells and the activity of the luciferase reporter was recorded in relative light units (RLU) per μl of virus stock.

### Illumina next generation sequencing

*Env* plasmids were sequenced with the Illumina MiSeq v2 50 cycles kit. For preprocessing and de novo assembly, CLC Genomics Server version 6.5 trim and mapping algorithms were used to trim 23 nucleotides from the 5′ and 3′ ends, followed by end quality trimming to obtain reads with an average Phred score >30 and free of ambiguous nucleotides. Contigs were generated using the de novo assembly algorithm with automatic bubble and word size parameters specified. HIV-1 contigs were then positively selected by aligning them to the HXB2 reference.

Patient plasma or primary virus isolates were sequenced with the Illumina MiSeq v2 300, 500 or 600 cycles kit. Reads were pre-processed using prinseq-lite.pl [82]. Prinseq-lite.pl allowed for quality trimming and removal of reads with an average Phred score <30 and ambiguous nucleotides. Overlapping read pairs were merged using Flash [83]. Alignments were generated using the Burrows-Wheeler Aligner BWA mem algorithm [84] with individual specific gp120 references. Individual specific gp120 references were derived by aligning the following to the Los Alamos HIV-1 HXB2 subtype B reference: (1) contigs obtained from *de novo* assembly—using the methods mentioned above; and (2) sample specific consensus sequences—called from CLC Genomic Workbench Extract Consensus Sequence algorithm for BWA mem alignments of sample specific reads (plasma or primary virus isolate) to a Zurich HIV-1 subtype B reference, with “Threshold = 100” and “Post-remove action = Split into separate sequences” specified. Haplotypes were generated for gp120 using QuasiRecomb [85] with the—conservative and—K 1-100 options specified. Haplotypes with frequencies >1 % and a Phred score >30 were retained for subsequent analysis.

### In vitro replicative capacity

To assess the in vitro replicative capacity on PBMCs, virus isolates were serially diluted in cell culture medium and 100 μl virus dilution was added in quadruplicates per dilution on 96-well plates containing 2 × 10^5^ stimulated CD8-depleted PBMCs in 100 μl culture medium per well. 50 μl culture supernatants were harvested and 50 μl fresh medium was added back on days 4, 5, 6, 7, 8, 10, 12 and 14 post infection (p.i.). At day 14 p.i. culture supernatants were assayed for p24 antigen by using an in-house p24 antigen enzyme-linked immunosorbent assay (ELISA) as described previously [86, 87] and the TCID_50_ was determined. Retrospectively, p24 antigen was measured by ELISA of the dilution containing the same virus inoculum (125 TCID_50_ per well as determined with day 14 p.i. supernatants of the same experiment) from all other harvesting days. Because the virus inoculum was not washed out after infection, the residual input p24 concentration was measured and subtracted from all test results.

To assess the in vitro replicative capacity on MDMs, 8 days after monocyte isolation 96-well plates containing 7 × 10^4^ MDMs per well were infected in triplicates with virus isolates at an MOI of 0.05, spinoculated onto MDMs (1200 g, 120′) and unbound virus was washed away. On day 1, 7 and 14 p.i. 50 μl culture supernatant was assayed for p24 antigen by ELISA.

To assess the in vitro replicative capacity in the presence of IFNα, 96-well plates containing 2 × 10^5^ stimulated CD8-depleted PBMCs in 100 μl of cell culture medium were pre-treated with 1000 U/ml of IFNα-2a (Roferon-A, Roche Pharmaceuticals) for 4 h at 37 °C. Virus isolates were then added at a TCID_50_ of 80 per well (as determined on the same pool of stimulated CD8-depleted PBMCs) in 100 μl cell culture medium to 6 wells of IFNα-2a treated and 6 wells of untreated PBMCs. Virus inoculum was washed out on day 1 p.i. Culture supernatants were harvested, assayed for p24 antigen by ELISA and culture wells fed with new medium on days 1, 3, 5, 7, 9, 11 and 13 p.i. In addition, on every day until day 8 (after that no new infection of PBMCs occurs) new IFNα-2a was added onto the culture wells. In this manner a final assay concentration of 1000 U/ml was maintained in the culture wells throughout the experiment as IFNα is known to have a short half-life.

As a measure of replicative capacity, we determined the AUC of the p24 production in ng/ml with following formula: AUC = (p24_2_ − p24_1_) * (T_2_ − T_1_)/ln(p24_2_/p24_1_). The sum of AUCs from successive time points then gave the total AUC.

### Neutralization assays

The neutralization activity of entry inhibitors and patient plasma against transmitter and recipient Env-pseudoviruses was evaluated in single-round infection assays using TZM-bl cells as described [81]. TZM-bl cells were seeded in 96-well plates (10,000 cells per well). Virus inoculum was chosen to yield an infectivity of 5000–20,000 RLU/well in the absence of inhibitors or plasma.

Pseudoviruses carrying the murine leukemia virus and the HIV-1 SF162 Envs were used as negative and positive controls for all plasma samples, respectively. The inhibitor concentration or reciprocal plasma dilution causing 50 % reduction in viral infectivity (IC_50_ or NT_50_) were derived by fitting pooled data from 2 independent experiments to the log inhibitor vs. response variable slope fit in GraphPad Prism. The MPN was defined as the average percent neutralization reached at the highest plasma concentration of 1:40. If 50 % inhibition or neutralization was not reached at the highest or lowest inhibitor or plasma concentration, a greater-than or less-than value was recorded and used for statistical tests.

### Entry kinetics assays

Entry kinetics was probed as previously described [51]. Briefly, TZM-bl cells were seeded in 96-well plates (20,000 cells per well). Env-pseudovirus stocks adjusted to 50,000 RLU in 100 μl culture medium were added per well and spinoculated for 70′ at 1200 g and 10 °C onto TZM-bl cells. Unbound virus was removed and infection was rendered permissive by addition of media warmed to 37 °C. Subsequently, the inhibitor T-20 was added in saturating concentrations (final assay concentration: 50 μg/ml) at defined time points post infection to block viral entry. 48 h after infection, the production of luciferase was quantified. The T-20 addition after 120′ value was set as 100 % and all other T-20 addition wells were normalized to it. The time until 50 % of virus entry was derived from entry kinetic curves.

### Cell–cell transmission and free virus infection

Cell–cell transmission and free virus infection was tested as described [52, 53]. 293-T cells were seeded in 12-well plates (1 × 10^5^ cells per well) and transfected with the *env* plasmids and the NLinGluc vector [88, 89] for cell–cell transmission or the NLluc-AM vector for free virus infection at a ratio of 1:3 using PEI.

To assess cell–cell transmission, 6 h after transfection the supernatant was removed and the 293-T cells were resuspended in 2 ml culture medium (without DEAE-dextran). 5 × 10^3^ transfected cells were seeded to 1.5 × 10^4^ A3.01-CCR5 cells per well (in triplicates per *env* plasmid). After 65 h incubation at 37 °C gaussia luciferase production was quantified (Renilla Luciferase Assay system, Promega).

To assess free virus transmission, 6 h after transfection the supernatant was replaced with 1 ml fresh culture medium. 48 h later, the virus-containing supernatant was harvested, cleared by centrifugation and stored at −80 °C. Subsequently, 50 μl virus was added onto 5 × 10^4^ A3.01-CCR5 cells per well in 96-well plates (in triplicates per *env* plasmid) in the presence of 10 μg/ml DEAE-dextran. After 65 h incubation at 37 °C firefly luciferase production was quantified.

### Statistical analysis

All statistical analyses were performed in GraphPad Prism 5 (GraphPad Software). Transmitter and recipient viruses were compared by the Wilcoxon matched-pairs signed rank test. When more than one Env-pseudovirus was measured per patient, the median value was taken for statistical tests. Correlation was performed by linear regression analysis. p value ≤0.05 was considered statistically significant.

## Additional files

10.1186/s12977-016-0299-0 Phylogenetic trees of *polymerase* and *envelope* sequences. (a) Maximum likelihood phylogenetic tree of *polymerase* (*pol*) sequences. Sequences of transmitters and recipients are indicated by red and blue dots, respectively. (b) Neighbor joining phylogenetic tree of *envelope* (*env*) single genome amplification (SGA) sequences (for T9 sequences are derived from full-length *env* clones after several SGA attempts failed). Bootstrap support is depicted on the respective node. For clarity, heights of black triangles depict number of sequences that clustered at branch end. Branch lengths are drawn to scale. HXB2 was used as an HIV-1 subtype B reference and reference strains of other HIV-1 subtypes were used as outgroup.
10.1186/s12977-016-0299-0 Viral load kinetics of transmission pairs. Viral load kinetics of transmitters (red) and recipients (blue). X axis illustrates time in years relative to the first patient visit/sample collection of recipients. Patient visits/sample collections are indicated by dots, viral load (VL) kinetics by solid lines and antiretroviral treatment (ART) periods as shaded area. Black arrows indicate the estimated date of transmission (EDT). Time points circled in black were used for experiments. A and B indicate time points when virus isolates, Env-pseudoviruses and single genome amplification (SGA) were generated for transmitters and recipients. Numbering from one to three indicates time points plasma samples were tested for neutralization experiments (Fig. 2).
10.1186/s12977-016-0299-0 Phylogenetic trees of gp120 sequences from transmission pairs. Neighbor joining trees of gp120 sequences from transmitters (red) and recipients (blue). Sequences derived from single genome amplification are displayed with filled circles and those inferred from cloning with open circles. Next generation sequencing haplotypes (frequency above 1 %) of plasma and primary virus isolates are indicated by filled and empty squares, respectively. X indicates the majority haplotype and the asterisks the consensus sequence. Not for all patients next generation sequencing data is present due to limitations in sample availability, failure of amplification or haplotype reconstruction. Triangles depict sequences that were used as Env-pseudoviruses for follow-up experiments. Branch lengths are drawn to scale and HIV-1 HXB2 was used as a subtype B reference.
10.1186/s12977-016-0299-0 Highlighter plots of transmitter and recipient Env sequences. The consensus of the single genome amplification (SGA) sequences of recipients were used as master sequences indicated on top and Env sequences of recipients (R) and transmitters (T) were aligned to it. Amino acid mismatches are illustrated by a colored bar. Sequences are derived from single genome amplification (SGA_X), cloning (cl_X) and from next generation sequencing of primary virus isolate and plasma virus. Note that haplotypes for primary virus isolates and plasma are only spanning gp120 and only those at a frequency above 1 % are depicted. Clones indicated in blue and red were used in follow up experiments for recipient and transmitter, respectively.
10.1186/s12977-016-0299-0 Sequence motifs in transmitter and recipient Env sequences.
10.1186/s12977-016-0299-0 Distances of transmitter and recipient *env* sequences to the most recent common ancestor (MRCA).
10.1186/s12977-016-0299-0 Maximal neutralization capacities of transmitter plasma samples. Maximal percent neutralization (MPN) of transmitter (red) and recipient (blue) Env-pseudoviruses by transmitter plasma from the closest time point to the EDT at the highest plasma concentration tested of 1:40. Line indicates 50 % neutralization and difference between median transmitter and recipient value was determined with a Wilcoxon matched-pairs signed rank test. No plasma sample to estimate neutralization capacity was available from transmitters T1 and T2; therefore they were excluded from this part of the analysis.
10.1186/s12977-016-0299-0 50 % neutralization titers of plasma samples from transmitters and recipients against transmitter and recipient Env-pseudoviruses.
10.1186/s12977-016-0299-0 50 % inhibitory concentrations for transmitter and recipient Env-pseudoviruses against a panel of entry inhibitors.
10.1186/s12977-016-0299-0 Transmitter and recipient viruses do not exhibit different replication fitness. Replicative capacity on PBMCs was measured over a 14 day period and different measures of replication fitness were compared between transmitter and recipient virus isolates. **(a)** Median area under the curve (AUC) until day 7. **(b)** Median absolute p24 concentration at day 7. **(c)** Median absolute p24 concentration at day 10. **(d)** Median p24 value maximally reached over the 14 day period. Values are medians of the two PBMC pools tested. Wilcoxon matched-pairs signed rank test was used to determine statistical significance.
10.1186/s12977-016-0299-0 Time to 50 % entry is comparable between transmitter and recipient viruses. Time to 50 % entry was determined for transmitter and recipient Env-pseudoviruses. Each data point represents one Env-pseudovirus according to the symbols of individual pairs on the right and for certain transmitters more than one Env-pseudovirus was tested. Data shown are means from three independent experiments each performed in duplicates.
10.1186/s12977-016-0299-0 Replicative capacity and resistance to IFNα are not associated. Linear regression analysis of **(a)** AUC in presence and in absence of IFNα in one representative experiment and **(b)** median AUC in absence of IFNα and median IFN resistance with r^2^ and p value depicted. Data for transmitters are indicated in red and for recipients in blue.
10.1186/s12977-016-0299-0 Statistical analysis of all experiments separated for transmission pairs with high and low diversity transmitters and for transmission pairs where recipient is closer to ancestral genotype, respectively.

## Abbreviations

AUC: area under the curve
EDT: estimated date of transmission
Env: envelope
HIV-1: human immunodeficiency virus type 1
IC_50_: 50 % inhibitory concentration
IFNα: interferon-α
MDM: monocyte-derived macrophage
MSM: men who have sex with men
MTF: male-to-female
NT_50_: 50 % neutralization titer
PBMC: peripheral blood mononuclear cell
PNGS: potential N-linked glycosylation site
Pol: polymerase
SGA: single genome amplification
SHCS: Swiss HIV Cohort Study
T/F: transmitted/founder
TCID_50_: 50 % tissue culture infectious dose
ZPHI: Zurich Primary HIV Infection study
