# Supplementary figures and images for: Tracing HIV-1 transmission: envelope traits of HIV-1 transmitter and recipient pairs
Source: Retrovirology. 2016 Sep 5;13(1):62. doi: 10.1186/s12977-016-0299-0 (PMC5011806; doi:10.1186/s12977-016-0299-0)

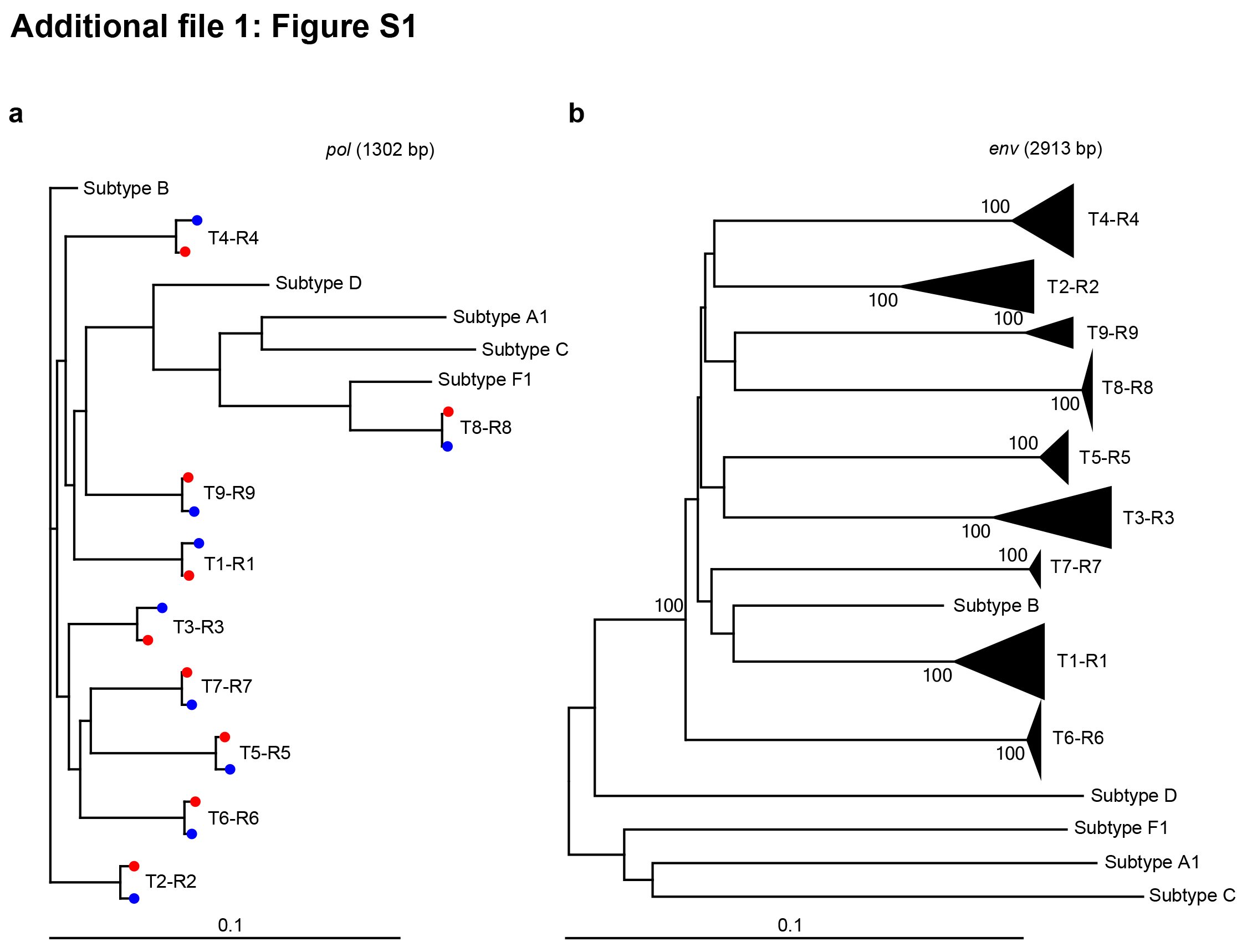

Supplement: Supplementary file 1 — 10.1186/s12977-016-0299-0 Phylogenetic trees of polymerase and envelope sequences. (a) Maximum likelihood phylogenetic tree of polymerase (pol) sequences. Sequences of transmitters and recipients are indicated by red and blue dots, respectively. (b) Neighbor joining phylogenetic tree of envelope (env) single genome amplification (SGA) sequences (for T9 sequences are derived from full-length env clones after several SGA attempts failed). Bootstrap support is depicted on the respective node. For clarity, heights of black triangles depict number of sequences that clustered at branch end. Branch lengths are drawn to scale. HXB2 was used as an HIV-1 subtype B reference and reference strains of other HIV-1 subtypes were used as outgroup. [file 12977_2016_299_MOESM1_ESM.tif]

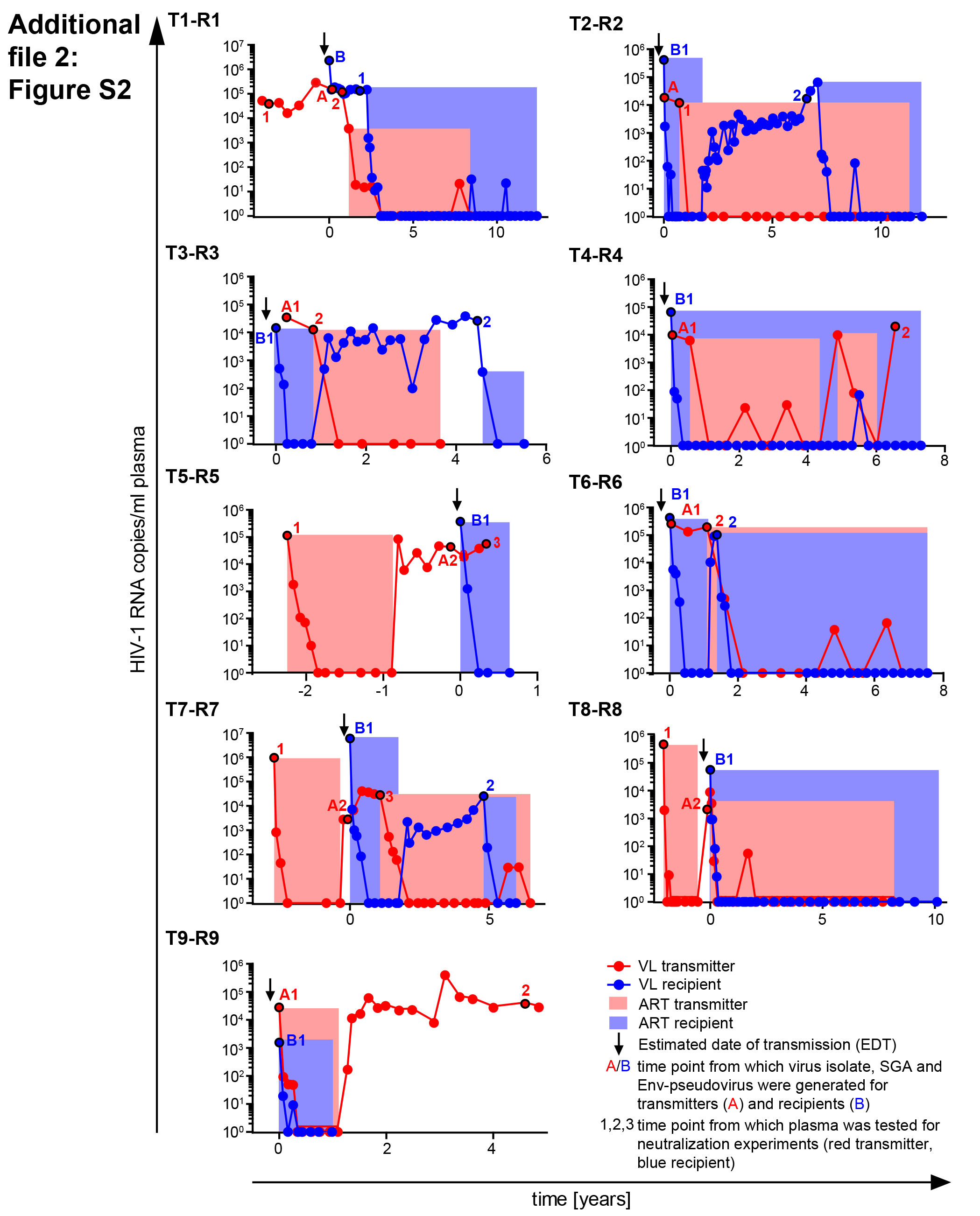

Supplement: Supplementary file 2 — 10.1186/s12977-016-0299-0 Viral load kinetics of transmission pairs. Viral load kinetics of transmitters (red) and recipients (blue). X axis illustrates time in years relative to the first patient visit/sample collection of recipients. Patient visits/sample collections are indicated by dots, viral load (VL) kinetics by solid lines and antiretroviral treatment (ART) periods as shaded area. Black arrows indicate the estimated date of transmission (EDT). Time points circled in black were used for experiments. A and B indicate time points when virus isolates, Env-pseudoviruses and single genome amplification (SGA) were generated for transmitters and recipients. Numbering from one to three indicates time points plasma samples were tested for neutralization experiments (Fig. 2). [file 12977_2016_299_MOESM2_ESM.tif]

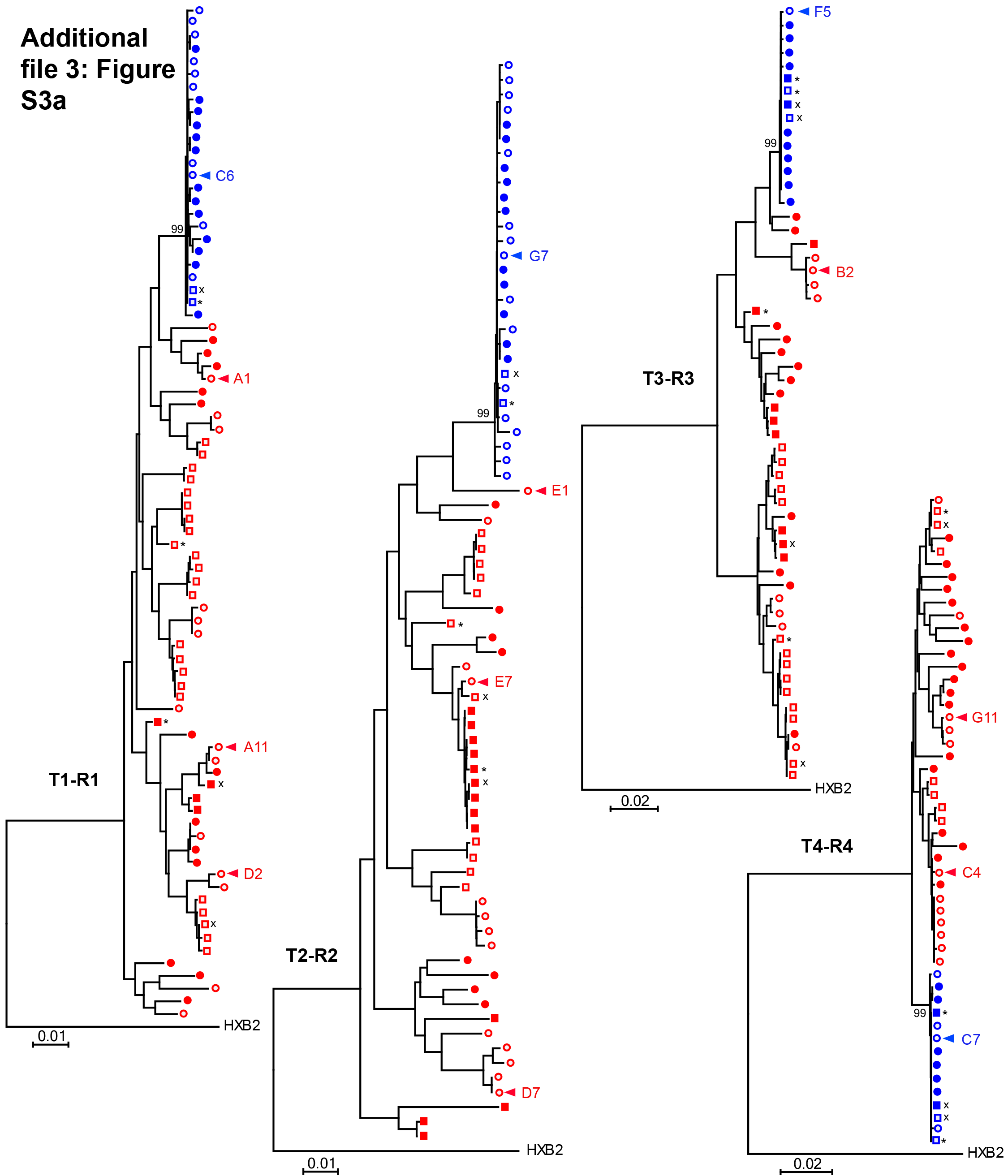

Supplement: Supplementary file 3 — 10.1186/s12977-016-0299-0 Phylogenetic trees of gp120 sequences from transmission pairs. Neighbor joining trees of gp120 sequences from transmitters (red) and recipients (blue). Sequences derived from single genome amplification are displayed with filled circles and those inferred from cloning with open circles. Next generation sequencing haplotypes (frequency above 1 %) of plasma and primary virus isolates are indicated by filled and empty squares, respectively. X indicates the majority haplotype and the asterisks the consensus sequence. Not for all patients next generation sequencing data is present due to limitations in sample availability, failure of amplification or haplotype reconstruction. Triangles depict sequences that were used as Env-pseudoviruses for follow-up experiments. Branch lengths are drawn to scale and HIV-1 HXB2 was used as a subtype B reference. [file 12977_2016_299_MOESM3_ESM.zip › Additional file 3/Additionalfile_3a.tif]

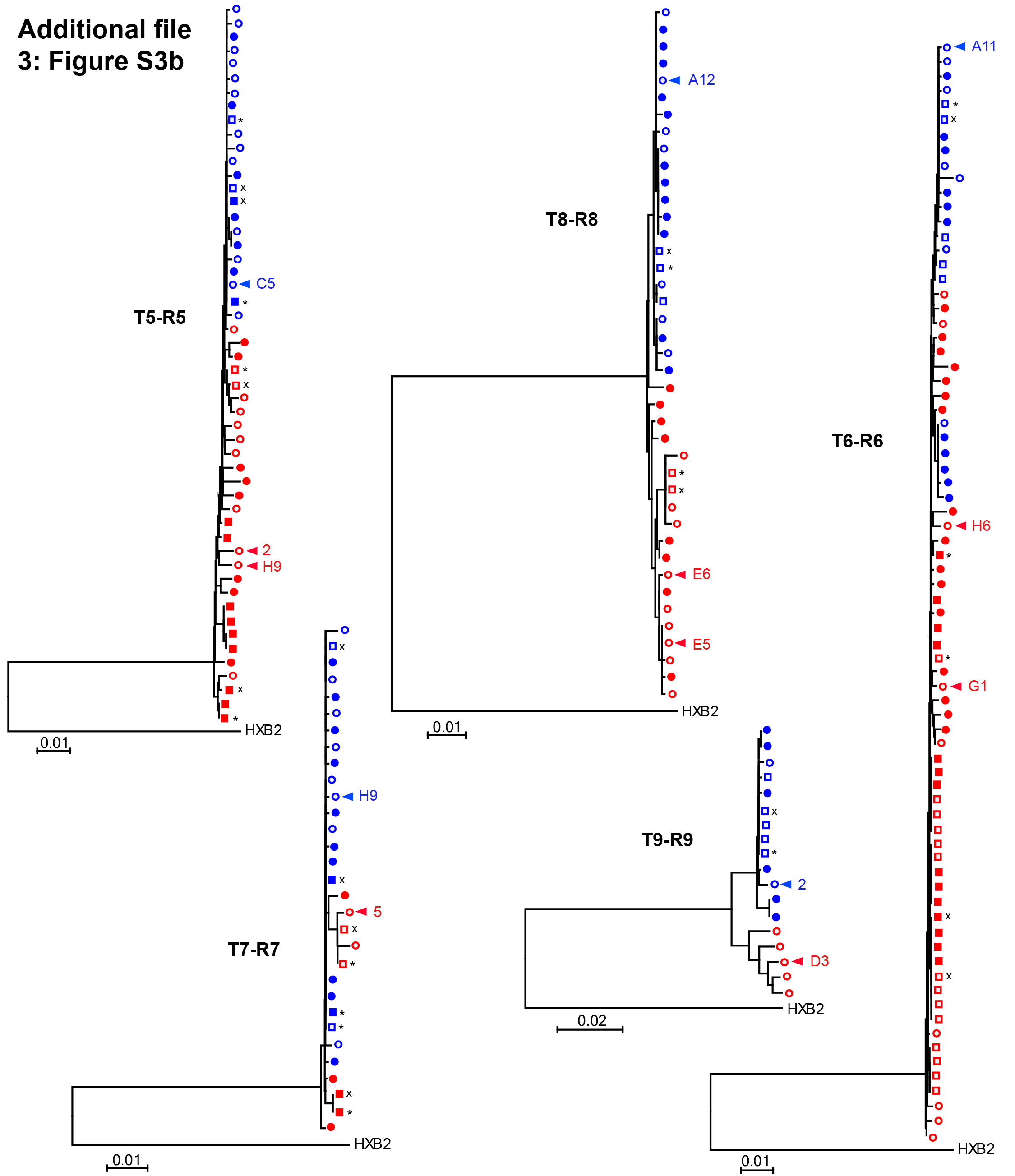

Supplement: Supplementary file 3 — 10.1186/s12977-016-0299-0 Phylogenetic trees of gp120 sequences from transmission pairs. Neighbor joining trees of gp120 sequences from transmitters (red) and recipients (blue). Sequences derived from single genome amplification are displayed with filled circles and those inferred from cloning with open circles. Next generation sequencing haplotypes (frequency above 1 %) of plasma and primary virus isolates are indicated by filled and empty squares, respectively. X indicates the majority haplotype and the asterisks the consensus sequence. Not for all patients next generation sequencing data is present due to limitations in sample availability, failure of amplification or haplotype reconstruction. Triangles depict sequences that were used as Env-pseudoviruses for follow-up experiments. Branch lengths are drawn to scale and HIV-1 HXB2 was used as a subtype B reference. [file 12977_2016_299_MOESM3_ESM.zip › Additional file 3/Additionalfile_3b.tif]

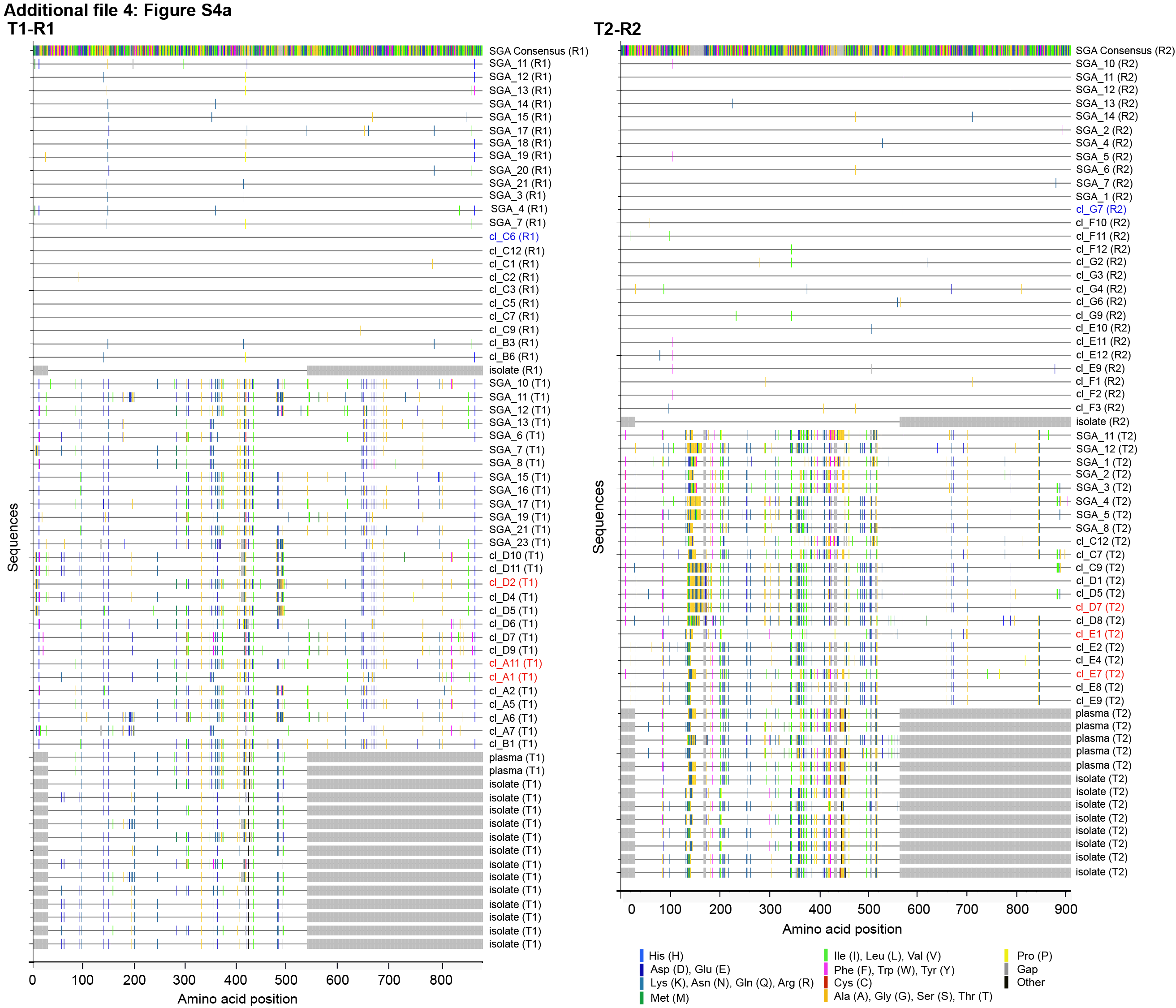

Supplement: Supplementary file 4 — 10.1186/s12977-016-0299-0 Highlighter plots of transmitter and recipient Env sequences. The consensus of the single genome amplification (SGA) sequences of recipients were used as master sequences indicated on top and Env sequences of recipients (R) and transmitters (T) were aligned to it. Amino acid mismatches are illustrated by a colored bar. Sequences are derived from single genome amplification (SGA_X), cloning (cl_X) and from next generation sequencing of primary virus isolate and plasma virus. Note that haplotypes for primary virus isolates and plasma are only spanning gp120 and only those at a frequency above 1 % are depicted. Clones indicated in blue and red were used in follow up experiments for recipient and transmitter, respectively. [file 12977_2016_299_MOESM4_ESM.zip › Additional 4/Additionalfile_4a.tif]

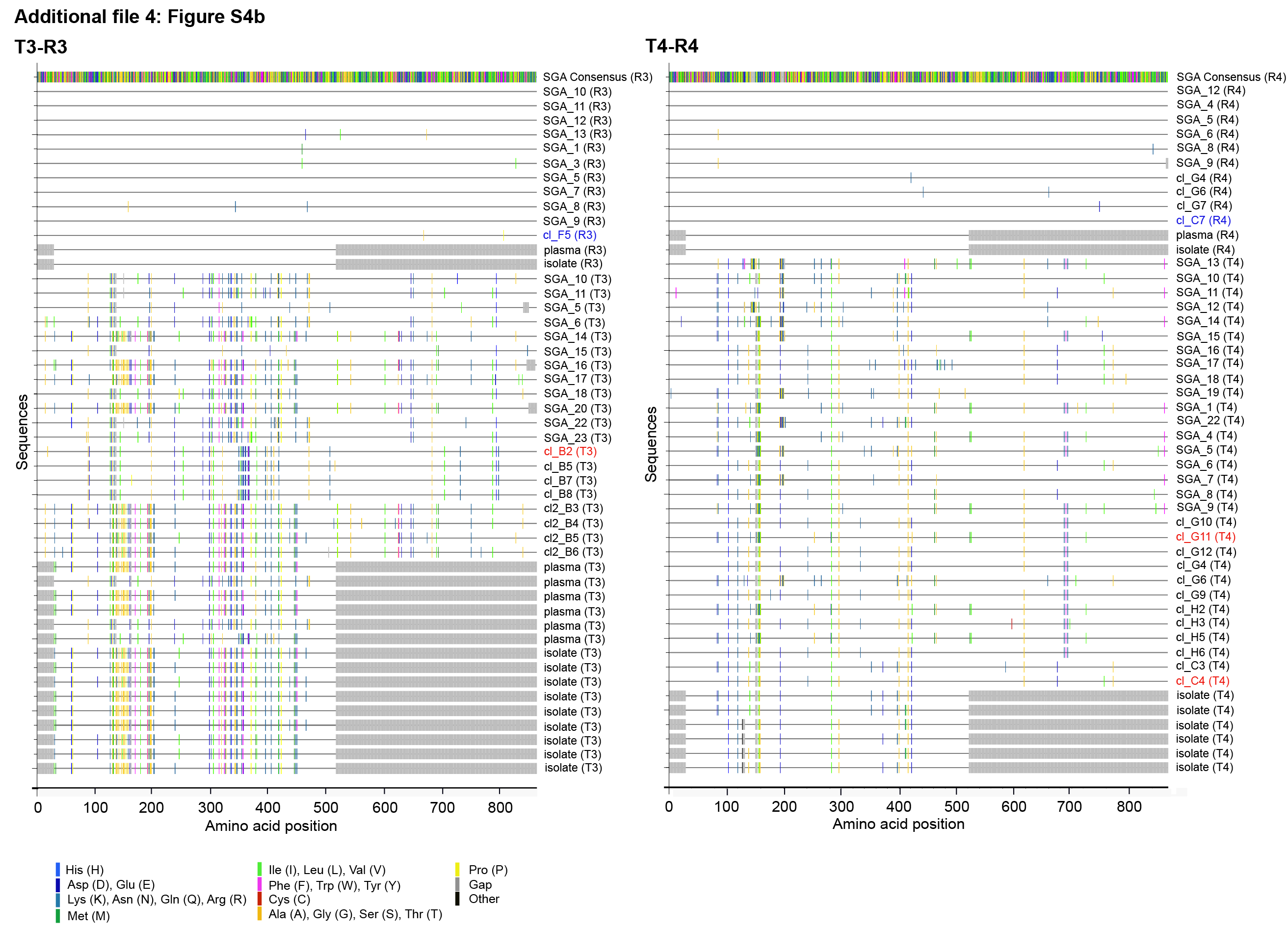

Supplement: Supplementary file 4 — 10.1186/s12977-016-0299-0 Highlighter plots of transmitter and recipient Env sequences. The consensus of the single genome amplification (SGA) sequences of recipients were used as master sequences indicated on top and Env sequences of recipients (R) and transmitters (T) were aligned to it. Amino acid mismatches are illustrated by a colored bar. Sequences are derived from single genome amplification (SGA_X), cloning (cl_X) and from next generation sequencing of primary virus isolate and plasma virus. Note that haplotypes for primary virus isolates and plasma are only spanning gp120 and only those at a frequency above 1 % are depicted. Clones indicated in blue and red were used in follow up experiments for recipient and transmitter, respectively. [file 12977_2016_299_MOESM4_ESM.zip › Additional 4/Additionalfile_4b.tif]

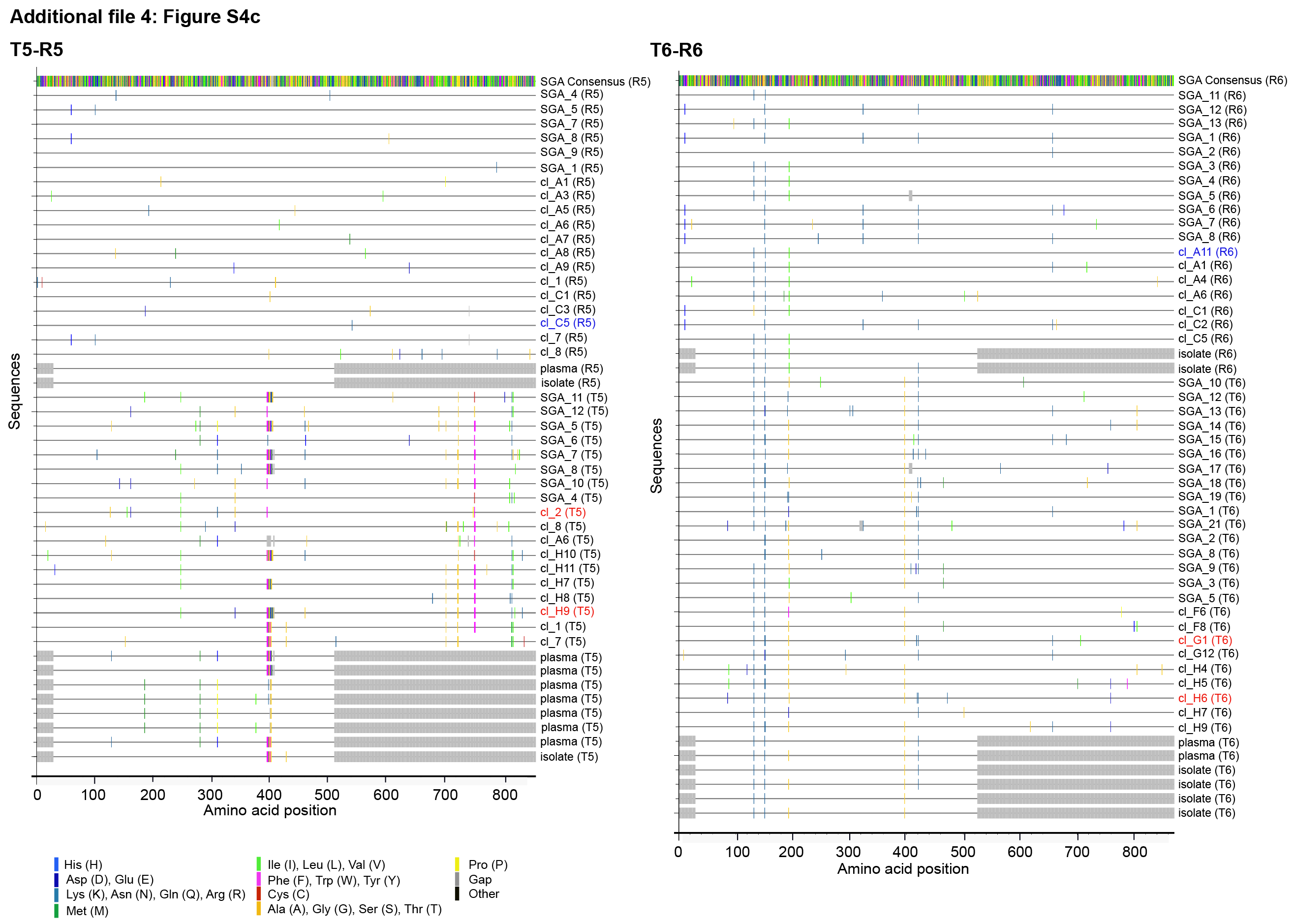

Supplement: Supplementary file 4 — 10.1186/s12977-016-0299-0 Highlighter plots of transmitter and recipient Env sequences. The consensus of the single genome amplification (SGA) sequences of recipients were used as master sequences indicated on top and Env sequences of recipients (R) and transmitters (T) were aligned to it. Amino acid mismatches are illustrated by a colored bar. Sequences are derived from single genome amplification (SGA_X), cloning (cl_X) and from next generation sequencing of primary virus isolate and plasma virus. Note that haplotypes for primary virus isolates and plasma are only spanning gp120 and only those at a frequency above 1 % are depicted. Clones indicated in blue and red were used in follow up experiments for recipient and transmitter, respectively. [file 12977_2016_299_MOESM4_ESM.zip › Additional 4/Additionalfile_4c.tif]

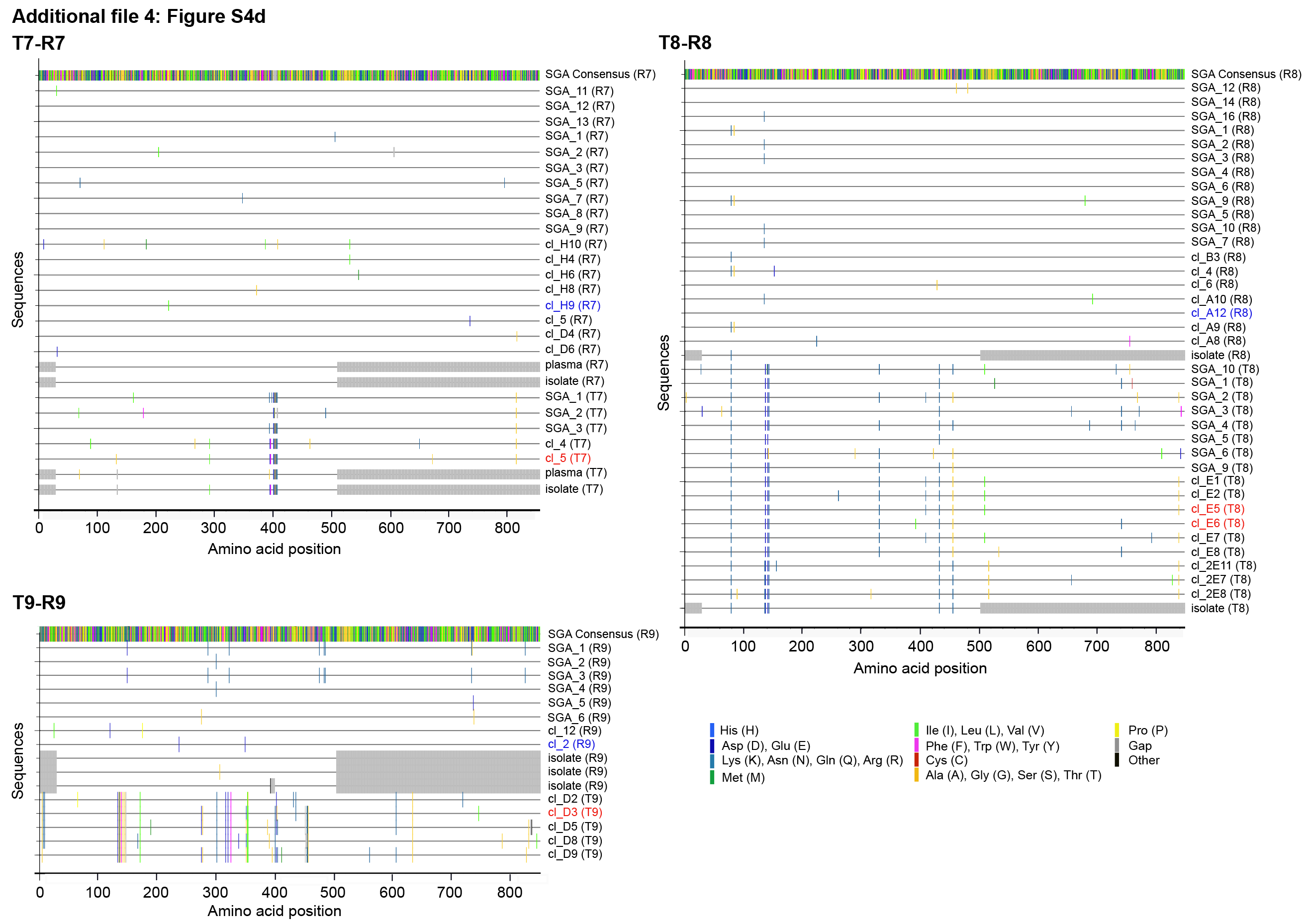

Supplement: Supplementary file 4 — 10.1186/s12977-016-0299-0 Highlighter plots of transmitter and recipient Env sequences. The consensus of the single genome amplification (SGA) sequences of recipients were used as master sequences indicated on top and Env sequences of recipients (R) and transmitters (T) were aligned to it. Amino acid mismatches are illustrated by a colored bar. Sequences are derived from single genome amplification (SGA_X), cloning (cl_X) and from next generation sequencing of primary virus isolate and plasma virus. Note that haplotypes for primary virus isolates and plasma are only spanning gp120 and only those at a frequency above 1 % are depicted. Clones indicated in blue and red were used in follow up experiments for recipient and transmitter, respectively. [file 12977_2016_299_MOESM4_ESM.zip › Additional 4/Additionalfile_4d.tif]

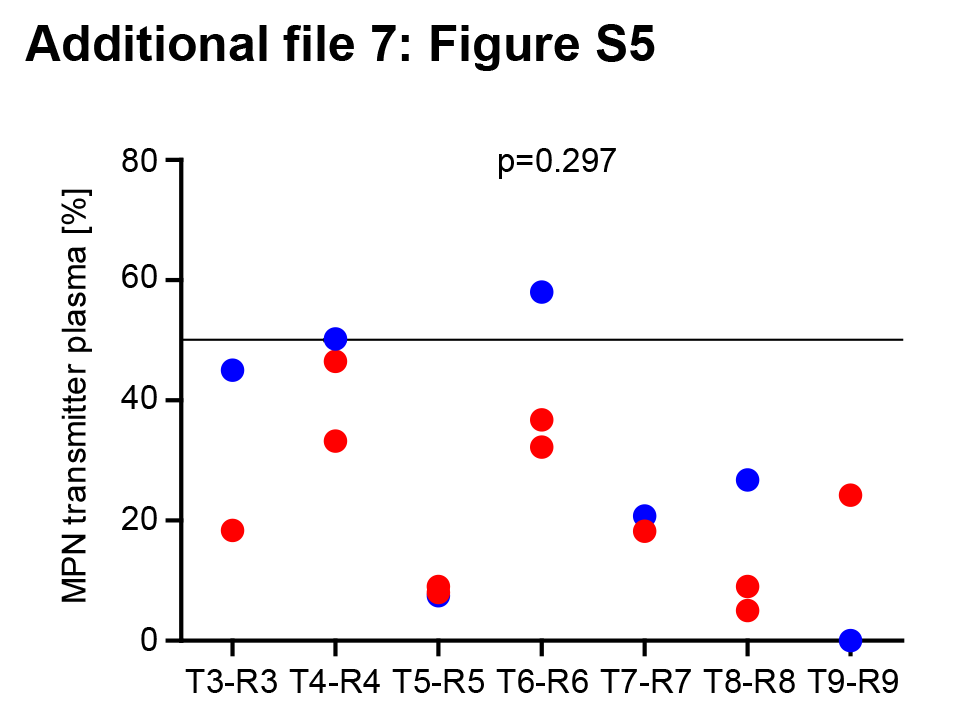

Supplement: Supplementary file 7 — 10.1186/s12977-016-0299-0 Maximal neutralization capacities of transmitter plasma samples. Maximal percent neutralization (MPN) of transmitter (red) and recipient (blue) Env-pseudoviruses by transmitter plasma from the closest time point to the EDT at the highest plasma concentration tested of 1:40. Line indicates 50 % neutralization and difference between median transmitter and recipient value was determined with a Wilcoxon matched-pairs signed rank test. No plasma sample to estimate neutralization capacity was available from transmitters T1 and T2; therefore they were excluded from this part of the analysis. [file 12977_2016_299_MOESM7_ESM.tif]

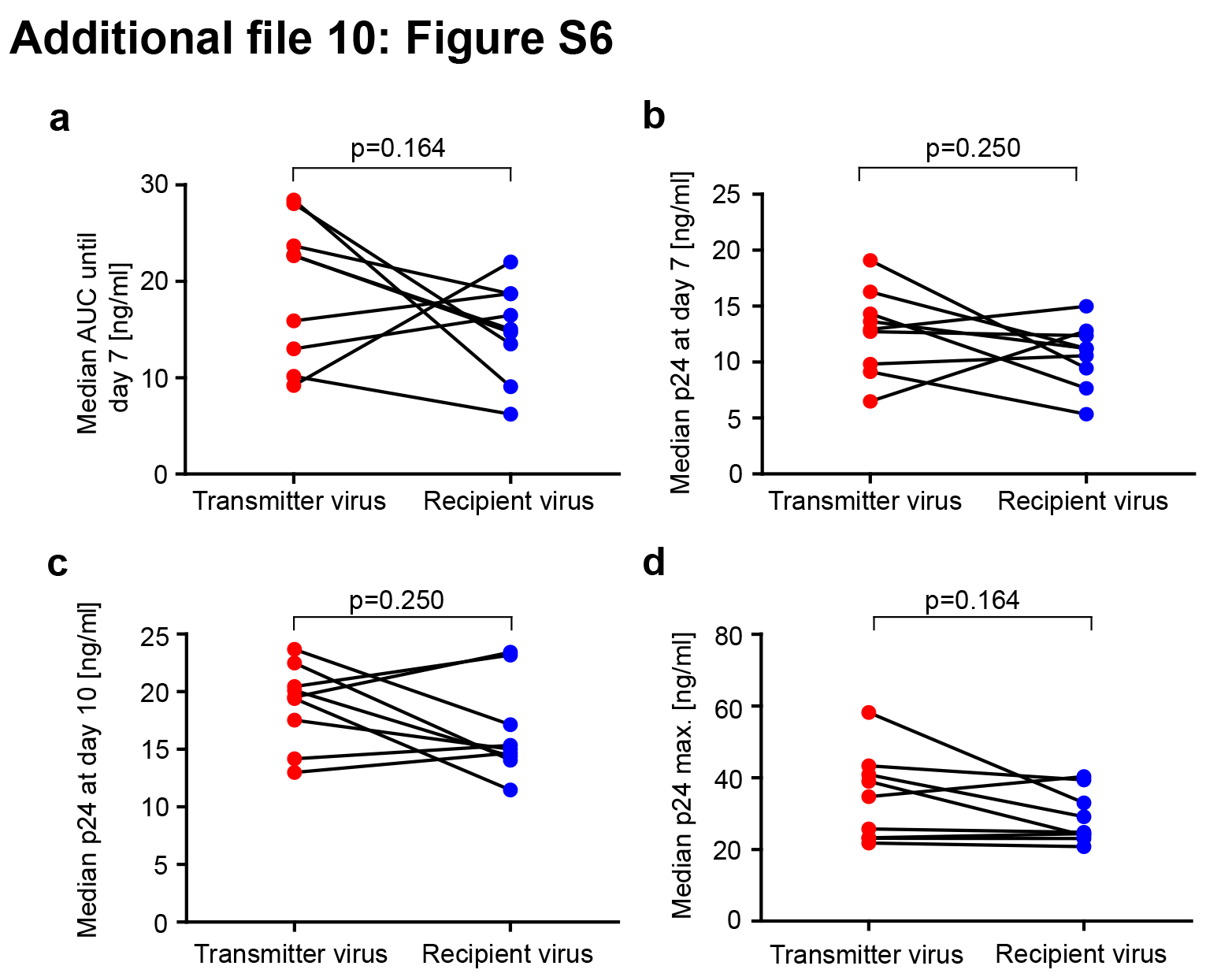

Supplement: Supplementary file 10 — 10.1186/s12977-016-0299-0 Transmitter and recipient viruses do not exhibit different replication fitness. Replicative capacity on PBMCs was measured over a 14 day period and different measures of replication fitness were compared between transmitter and recipient virus isolates. (a) Median area under the curve (AUC) until day 7. (b) Median absolute p24 concentration at day 7. (c) Median absolute p24 concentration at day 10. (d) Median p24 value maximally reached over the 14 day period. Values are medians of the two PBMC pools tested. Wilcoxon matched-pairs signed rank test was used to determine statistical significance. [file 12977_2016_299_MOESM10_ESM.tif]

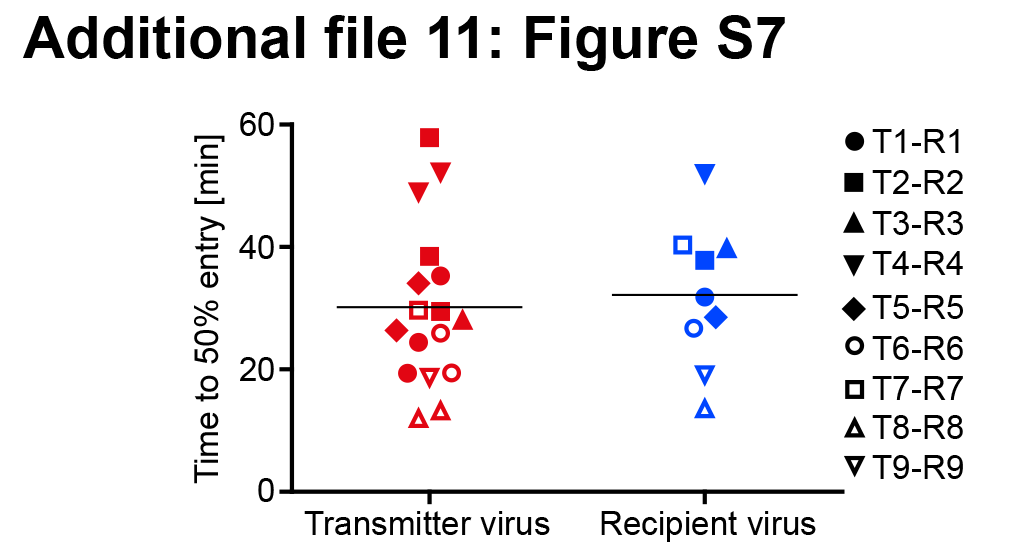

Supplement: Supplementary file 11 — 10.1186/s12977-016-0299-0 Time to 50 % entry is comparable between transmitter and recipient viruses. Time to 50 % entry was determined for transmitter and recipient Env-pseudoviruses. Each data point represents one Env-pseudovirus according to the symbols of individual pairs on the right and for certain transmitters more than one Env-pseudovirus was tested. Data shown are means from three independent experiments each performed in duplicates. [file 12977_2016_299_MOESM11_ESM.tif]

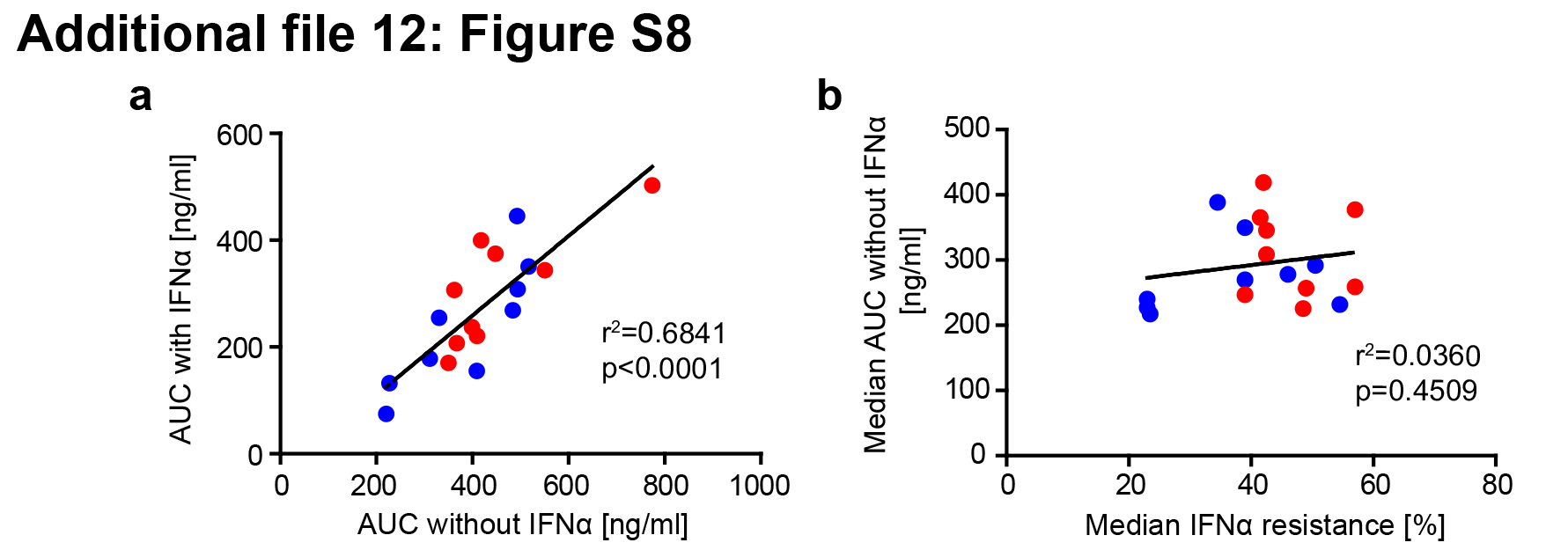

Supplement: Supplementary file 12 — 10.1186/s12977-016-0299-0 Replicative capacity and resistance to IFNα are not associated. Linear regression analysis of (a) AUC in presence and in absence of IFNα in one representative experiment and (b) median AUC in absence of IFNα and median IFN resistance with r2 and p value depicted. Data for transmitters are indicated in red and for recipients in blue. [file 12977_2016_299_MOESM12_ESM.tif]
