# Supplementary material for: Tracing HIV-1 transmission: envelope traits of HIV-1 transmitter and recipient pairs
Source: Retrovirology. 2016 Sep 5;13(1):62. doi: 10.1186/s12977-016-0299-0 (PMC5011806; doi:10.1186/s12977-016-0299-0)
Supplement: Supplementary file 5 — 10.1186/s12977-016-0299-0 Sequence motifs in transmitter and recipient Env sequences. [file 12977_2016_299_MOESM5_ESM.docx]

**Additional file 5: Table S1. Sequence motifs in transmitter and recipient Env sequences.**

| **Pair** | **No. of *env* sequences** | | **V1V2** | | | | | | **V4** | | | | | | **H12** | | **PNGS 413-415** | | **α4β7 binding site** | |
| --- | --- | --- | --- | --- | --- | --- | --- | --- | --- | --- | --- | --- | --- | --- | --- | --- | --- | --- | --- | --- |
|  |  | | **Length (amino acid)** | | | **PNGS** | | | **Length (amino acid)** | | | **PNGS** | | |  | |  | |  | |
|  | **T** | **R** | **T** | **R** | **p** | **T** | **R** | **p** | **T** | **R** | **p** | **T** | **R** | **p** | **T** | **R** | **T** | **R** | **T** | **R** |
| **T1-R1** | 28 | 23 | 77  (74-79) | 77  (75-77) | 0.987 | 8  (7-9) | 8  (7-8) | 0.202 | 35  (30-38) | 33  (33-33) | <0.001 | 6  (4-6) | 5  (5-5) | <0.001 | + (in 26/28) | - (in 21/23) | - | - | LDV | LDV |
| **T2-R2** | 21 | 27 | 67  (58-85) | 61  (61-61) | <0.001 | 5  (4-10) | 5  (5-5) | 0.003 | 29  (29-55) | 35  (35-35) | 0.058 | 3  (3-6) | 4  (4-4) | 0.009 | + | + | - | - | LD(V/L) | LDI |
| **T3-R3** | 20 | 11 | 70  (69-75) | 72  (72-72) | 0.356 | 7  (5-8) | 8  (8-8) | <0.001 | 32  (30-33) | 32  (32-32) | 0.196 | 6  (4-6) | 5  (5-5) | 0.008 | + | + | + (in 18/20) | - | LDI | LDI |
| **T4-R4** | 30 | 10 | 64  (62-76) | 66  (66-66) | 0.076 | 6  (5-8) | 7  (7-7) | 0.013 | 33  (33-33) | 33  (33-33) | n.d. | 4  (4-5) | 4  (4-4) | 0.092 | + | + | - | - | LDI | LDI |
| **T5-R5** | 18 | 19 | 70  (70-70) | 70  (70-70) | n.d. | 7  (6-7) | 7  (7-7) | 0.072 | 30.5  (21-34) | 30  (30-30) | 0.003 | 6  (2-7) | 6  (5-6) | 0.598 | + | + | + | + | LDV | LDV |
| **T6-R6** | 25 | 18 | 78  (77-78) | 78  (78-78) | 0.423 | 7  (7-8) | 7  (7-7) | 0.049 | 38  (32-38) | 38  (32-38) | 0.840 | 6  (5-7) | 5  (4-5) | <0.001 | + | + | - | - | LDI | LDI |
| **T7-R7** | 5 | 18 | 68  (68-68) | 68  (68-68) | n.d. | 7  (6-7) | 7  (7-7) | 0.073 | 30  (25-30) | 24  (24-24) | <0.001 | 3  (2-3) | 2  (1-2) | <0.001 | + | + | - | - | (L/F)DI | LDI |
| **T8-R8** | 17 | 19 | 59  (59-59) | 59  (59-59) | n.d. | 5  (4-5) | 5  (4-5) | 0.257 | 32  (32-32) | 32  (32-32) | n.d. | 3  (3-3) | 3  (3-3) | n.d. | - | - | - | - | TDV | TDV |
| **T9-R9** | 5^a^ | 8 | 62  (62-62) | 63  (63-63) | <0.001 | 6  (6-6) | 6  (6-6) | n.d. | 31  (31-31) | 31  (31-31) | n.d. | 4  (4-5) | 4  (4-4) | 0.080 | - | - | - (in 3/5) | - | LDV | (P/L)DV |
| **overall** |  |  | 68  (59-78) | 68  (59-78) | 0.854 | 7  (5-8) | 7  (5-8) | 0.346 | 32  (29-38) | 32  (24-38) | 0.713 | 4  (3-6) | 4  (2-6) | 0.233 |  |  |  |  |  |  |

Median (range) for length and potential N-linked glycosylation sites (PNGS) of V1V2 and V4 in individual pairs are shown and statistical significance between transmitter (T) and recipient (R) sequences was calculated with the Mann-Whitney U test and p values are indicated in each row (n.d.=not determined). Overall median (range) of all transmission pair medians are shown at the bottom and median of all transmitters and recipients was compared with the Wilcoxon matched-pairs signed rank test and p values are indicated. The presence (+) or absence (-) of Histidine at position 12 (H12) and a PNGS at position 413-415 and if not present/absent in all sequences the respective numbers are indicated. The V2 tripeptide motif enabling binding to the integrin α4β7 is indicated for transmitter and recipient sequences.

^a^ *S*equences of T9 are derived from full-length *env* clones after several SGA attempts failed.
