# Supplementary material for: Tracing HIV-1 transmission: envelope traits of HIV-1 transmitter and recipient pairs
Source: Retrovirology. 2016 Sep 5;13(1):62. doi: 10.1186/s12977-016-0299-0 (PMC5011806; doi:10.1186/s12977-016-0299-0)
Supplement: Supplementary file 6 — 10.1186/s12977-016-0299-0 Distances of transmitter and recipient env sequences to the most recent common ancestor (MRCA). [file 12977_2016_299_MOESM6_ESM.docx]

**Additional file 6: Table S2. Distances of transmitter and recipient *env* sequences to the most recent common ancestor (MRCA).**

| **Distances to MRCA** | | |
| --- | --- | --- |
|  | Transmitters (%) | Recipients (%) |
| T1-R1 | 2.41 | 2.12 |
| T2-R2 | 3.71 | 3.37 |
| T3-R3 | 2.6 | 2.55 |
| T4-R4 | 1.49 | 1.58 |
| T5-R5 | 0.75 | 0.81 |
| T6-R6 | 0.49 | 0.28 |
| T7-R7 | 0.34 | 0.09 |
| T8-R8 | 0.44 | 0.52 |
| T9-R9 | 1.2 | 0.95 |
