# Supplementary material for: Tracing HIV-1 transmission: envelope traits of HIV-1 transmitter and recipient pairs
Source: Retrovirology. 2016 Sep 5;13(1):62. doi: 10.1186/s12977-016-0299-0 (PMC5011806; doi:10.1186/s12977-016-0299-0)
Supplement: Supplementary file 8 — 10.1186/s12977-016-0299-0 50 % neutralization titers of plasma samples from transmitters and recipients against transmitter and recipient Env-pseudoviruses. [file 12977_2016_299_MOESM8_ESM.docx]

**Additional file 8: Table S3. 50% neutralization titers of plasma samples from transmitters and recipients against transmitter and recipient Env-pseudoviruses.**

| **Virus** | **Plasma T1 -1216d** | Plasma T1 -569d | Plasma T1 147d | **Plasma T1 363d** | Plasma R1 49d | **Plasma R1 667d** |
| --- | --- | --- | --- | --- | --- | --- |
| **T1 (D2)** | <40 | <40 | 45 | 202 | <40 | 614 |
| **T1 (A11)** | <40 | <40 | 95 | 251 | <40 | 473 |
| **T1 (A1)** | <40 | <40 | 141 | 1042 | <40 | 5912 |
| **R1** | <40 | <40 | 166 | 1907 | 119 | 6922 |
|  |  |  |  |  |  |  |
|  | **Plasma T2 253d** | **Plasma R2** | Plasma R2 814d | Plasma R2 1592d | **Plasma R2 2402d** |  |
| **T2 (E1)** | 53 | <40 | <40 | <40 | 42 |  |
| **T2 (D7)** | 130 | <40 | <40 | 52 | 208 |  |
| **T2 (E7)** | 235 | <40 | <40 | 42 | 258 |  |
| **R2** | 124 | <40 | 2743 | 1705 | 851 |  |
|  |  |  |  |  |  |  |
|  | **Plasma T3** | **Plasma T3 216d** | **Plasma R3** | Plasma R3 607d | Plasma R3 1205d | **Plasma R3 1632d** |
| **T3** | <40 | <40 | <40 | <40 | 168 | 354 |
| **R3** | <40 | <40 | <40 | 249 | 2684 | 4057 |
|  |  |  |  |  |  |  |
|  | **Plasma T4** | Plasma T4 188d | Plasma T4 1763d | **Plasma T4 2378d** | **Plasma R4** |  |
| **T4 (C4)** | <40 | 56 | 621 | 675 | <40 |  |
| **T4 (G11)** | <40 | 55 | 758 | 759 | <40 |  |
| **R4** | <40 | 120 | 582 | 969 | <40 |  |
|  |  |  |  |  |  |  |
|  | **Plasma T5 -774d** | Plasma T5 -161d | **Plasma T5** | **Plasma T5 169d** | **Plasma R5** |  |
| **T5 (H9)** | <40 | <40 | <40 | 108 | <40 |  |
| **T5 (2)** | <40 | <40 | <40 | 143 | <40 |  |
| **R5** | <40 | <40 | <40 | <40 | <40 |  |
|  |  |  |  |  |  |  |
|  | **Plasma T6** | Plasma T6 180d | **Plasma T6 383d** | **Plasma R6** | **Plasma R6 504d** |  |
| **T6 (G1)** | <40 | 82 | 106 | <40 | <40 |  |
| **T6 (H6)** | <40 | 55 | 45 | <40 | <40 |  |
| **R6** | <40 | 466 | 349 | <40 | <40 |  |
|  |  |  |  |  |  |  |
|  | **Plasma T7 -966d** | **Plasma T7** | **Plasma T7 425d** | **Plasma R7** | Plasma R7 902d | **Plasma R7 1757d** |
| **T7** | <40 | <40 | 648 | <40 | 63 | 121 |
| **R7** | <40 | <40 | 3680 | <40 | 1280 | 2912 |
|  |  |  |  |  |  |  |
|  | **Plasma T8 -706d** | **Plasma T8** | **Plasma R8** |  |  |  |
| **T8 (E5)** | <40 | <40 | <40 |  |  |  |
| **T8 (E6)** | <40 | <40 | <40 |  |  |  |
| **R8** | <40 | <40 | <40 |  |  |  |
|  |  |  |  |  |  |  |
|  | **Plasma T9** | Plasma T9 673d | Plasma T9 1123d | **Plasma T9 1679d** | **Plasma R9** |  |
| **T9** | <40 | 1032 | 1953 | 2079 | <40 |  |
| **R9** | <40 | 285 | 344 | 379 | <40 |  |

The 50% neutralization titer (NT_50_), i.e., the reciprocal plasma dilution yielding 50% neutralization, of transmitter (T) and recipient (R) plasma against transmitter and recipient Env-pseudoviruses is depicted. Plasma samples from transmitters and recipients are indicated on top. Plasma samples shaded in grey are from the closest time point to the estimated date of transmission (EDT) and plasma samples from before or after the EDT with respective time span in days are shaded in white. Plasma samples written in bold were used for Fig 2. On the left Env-pseudoviruses of transmitters and recipients are depicted. Green indicates NT_50_ values <40, yellow between 40 and 1000 and red >1000. Neutralization titers were derived from 2 independent experiments each performed in duplicates.
