# Supplementary material for: Tracing HIV-1 transmission: envelope traits of HIV-1 transmitter and recipient pairs
Source: Retrovirology. 2016 Sep 5;13(1):62. doi: 10.1186/s12977-016-0299-0 (PMC5011806; doi:10.1186/s12977-016-0299-0)
Supplement: Supplementary file 9 — 10.1186/s12977-016-0299-0 50 % inhibitory concentrations for transmitter and recipient Env-pseudoviruses against a panel of entry inhibitors. [file 12977_2016_299_MOESM9_ESM.docx]

| **Additional file 9: Table S4. 50% inhibitory concentrations for transmitter and recipient Env-pseudoviruses against a panel of entry inhibitors.** | | | | | | | | | | | | | |  |
| --- | --- | --- | --- | --- | --- | --- | --- | --- | --- | --- | --- | --- | --- | --- |
| **Virus** | **CD4-IgG2** | **VRC01** | **b12** | **b6** | **2G12** | **PGT121** | **PGT128** | **1.79** | **T-20** | **2F5** | **4E10** | **DARPin 27.2** | **Maraviroc** |  |
| **T1 (D2)** | >20 | 3.2 | 0.64 | >25 | >25 | >25 | 0.011 | >25 | 0.009 | 5.2 | 10.7 | 0.016 | 0.0022 |  |
| **T1 (A11)** | 2.3 | 0.9 | 0.29 | >25 | >25 | >25 | 0.008 | >25 | 0.007 | 6.1 | 7.6 | 0.024 | 0.0043 |  |
| **T1 (A1)** | >20 | 1.3 | 0.28 | >25 | >25 | >25 | 0.007 | >25 | 0.012 | 1.2 | 2.3 | 0.006 | 0.0007 |  |
| **R1** | 4.5 | 1.8 | 0.24 | >25 | >25 | 6.416 | 0.008 | >25 | 0.009 | 2.9 | 6.3 | 0.009 | 0.0027 |  |
| **T2 (E1)** | 2.2 | >25 | >25 | >25 | >25 | 1.399 | 0.207 | >25 | 0.106 | 3.0 | >25 | 0.013 | 0.0028 |  |
| **T2 (D7)** | 2.1 | >25 | >25 | >25 | >25 | >25 | >25 | >25 | 0.053 | 6.7 | >25 | 0.015 | 0.0028 |  |
| **T2 (E7)** | 0.2 | >25 | >25 | >25 | >25 | 2.613 | 0.342 | >25 | 0.043 | 1.6 | 6.1 | 0.016 | 0.0019 |  |
| **R2** | 0.9 | 2.4 | >25 | >25 | >25 | >25 | 0.550 | >25 | 0.056 | 14.3 | >25 | 0.019 | 0.0013 |  |
| **T3** | >20 | 5.6 | >25 | >25 | >25 | >25 | >25 | >25 | 0.060 | 2.5 | >25 | 0.009 | 0.0011 |  |
| **R3** | >20 | 6.8 | >25 | >25 | >25 | 0.119 | 1.906 | >25 | 0.045 | >25 | 10.0 | 0.007 | 0.0008 |  |
| **T4 (C4)** | >20 | 6.9 | 9.27 | >25 | 23.2 | 0.073 | 0.007 | >25 | 0.023 | >25 | 7.5 | 0.013 | 0.0033 |  |
| **T4 (G11)** | >20 | 4.4 | >25 | >25 | 22.0 | 0.045 | 0.007 | >25 | 0.027 | >25 | >25 | 0.014 | 0.0029 |  |
| **R4** | 19.8 | 13.7 | 8.08 | >25 | >25 | 0.452 | 0.009 | >25 | 0.015 | >25 | >25 | 0.015 | 0.0068 |  |
| **T5 (H9)** | >20 | >25 | >25 | >25 | 5.0 | 0.015 | 0.024 | >25 | 0.570 | 3.2 | >25 | 0.012 | 0.0030 |  |
| **T5 (2)** | 9.8 | >25 | >25 | >25 | 10.2 | 0.011 | 0.023 | >25 | 0.338 | 11.9 | >25 | 0.016 | 0.0051 |  |
| **R5** | >20 | >25 | >25 | >25 | 9.1 | 0.022 | 0.034 | >25 | 0.323 | 7.7 | >25 | 0.010 | 0.0031 |  |
| **T6 (G1)** | 4.2 | 1.2 | 0.60 | >25 | >25 | >25 | >25 | >25 | 0.296 | 3.6 | 10.5 | 0.014 | 0.0060 |  |
| **T6 (H6)** | 2.0 | 1.4 | 0.30 | >25 | >25 | >25 | >25 | >25 | 0.406 | 1.9 | 8.4 | 0.010 | 0.0043 |  |
| **R6** | 8.2 | 0.7 | 0.04 | >25 | >25 | >25 | >25 | >25 | 0.315 | 3.1 | 7.5 | 0.009 | 0.0038 |  |
| **T7** | 1.3 | 6.5 | 1.77 | >25 | 1.5 | 0.042 | 0.005 | >25 | 0.015 | 1.1 | >25 | 0.013 | 0.0027 |  |
| **R7** | 7.6 | 9.3 | >25 | >25 | 1.4 | 0.020 | 0.004 | >25 | 0.008 | 1.5 | 4.6 | 0.007 | 0.0025 |  |
| **T8 (E5)** | >20 | 0.3 | >25 | >25 | >25 | 0.003 | 0.005 | >25 | 0.395 | >25 | 12.6 | 0.010 | 0.0024 |  |
| **T8 (E6)** | 14.5 | 0.2 | >25 | >25 | >25 | 0.003 | 0.006 | >25 | 0.335 | >25 | 11.0 | 0.011 | 0.0063 |  |
| **R8** | >20 | 0.3 | >25 | >25 | >25 | 0.002 | 0.006 | >25 | 0.283 | >25 | 12.8 | 0.009 | 0.0080 |  |
| **T9** | >20 | 0.5 | >25 | >25 | 10.7 | 0.005 | 0.006 | >25 | 0.215 | 6.7 | 6.3 | 0.010 | 0.0052 |  |
| **R9** | >20 | >25 | >25 | >25 | 2.2 | 0.034 | 0.015 | >25 | 0.335 | 8.1 | >25 | 0.011 | 0.0102 |  |

50% inhibitory concentrations (IC_50_) in μg/ml. Values above the maximal inhibitor concentration tested are indicated with a greater-than value. Note that for certain transmitters more than one Env-pseudovirus was tested. Values are from 2 independent experiments each performed in duplicates.
