# Supplementary material for: Tracing HIV-1 transmission: envelope traits of HIV-1 transmitter and recipient pairs
Source: Retrovirology. 2016 Sep 5;13(1):62. doi: 10.1186/s12977-016-0299-0 (PMC5011806; doi:10.1186/s12977-016-0299-0)
Supplement: Supplementary file 13 — 10.1186/s12977-016-0299-0 Statistical analysis of all experiments separated for transmission pairs with high and low diversity transmitters and for transmission pairs where recipient is closer to ancestral genotype, respectively. [file 12977_2016_299_MOESM13_ESM.docx]

**Additional file 13: Table S5. Statistical analysis of all experiments separated for transmission pairs with high and low diversity transmitters and for transmission pairs where recipient is closer to ancestral genotype, respectively.**

**Threshold for separation into high and low diversity transmitters 1%**

| Experiment | p value for high (T1-R1, T2-R2, T3-R3, T4-R4, T9-R9) | p value for low (T5-R5, T6-R6, T7-R7, T8-R8) |
| --- | --- | --- |
| V1V2 length | 0.854 | not determined (same value) |
| V1V2 glycosylation | 0.346 | not determined (same value) |
| V4 length | 1.000 | 0.500 |
| V4 glycosylation | 0.773 | 0.346 |
| Maximal percent neutralization transmitter plasma | 0.750 | 0.250 |
| IC_50_ CD4**-**IgG2 | 0.250 | 0.125 |
| IC_50_ VRC01 | 0.438 | 0.750 |
| IC_50_ b12 | 0.500 | 1.000 |
| IC_50_ 2G12 | 1.000 | 1.000 |
| IC_50_ PGT121 | 0.645 | 0.621 |
| IC_50_ PGT128 | 0.813 | 0.750 |
| IC_50_ T20 | 1.000 | 0.125 |
| IC_50_ 2F5 | 0.375 | 0.250 |
| IC_50_ 4E10 | 0.875 | 0.500 |
| IC_50_ DARPin 27.2 | 1.000 | 0.125 |
| IC_50_ Maraviroc | 0.438 | 0.875 |
| Median AUC PBMCs | 0.125 | 0.875 |
| Median AUC MDMs | 1.000 | 0.250 |
| Median IFNα resistance | 0.188 | 0.125 |
| Median time to 50% entry | 0.188 | 0.375 |
| Cell-cell transmission | 0.438 | 0.125 |
| Free virus infection | 1.000 | 0.125 |

**Threshold for separation into high and low diversity transmitters 0.6%**

| Experiment | p value for high (T1-R1, T2-R2, T3-R3, T4-R4, T5-R5, T9-R9) | p value for low (T6-R6, T7-R7, T8-R8) |
| --- | --- | --- |
| V1V2 length | 0.854 | not determined (same value) |
| V1V2 glycosylation | 0.346 | not determined (same value) |
| V4 length | 1.000 | 1.000 |
| V4 glycosylation | 0.773 | 0.346 |
| Maximal percent neutralization transmitter plasma | 0.875 | 0.250 |
| IC_50_ CD4**-**IgG2 | 0.625 | 0.250 |
| IC_50_ VRC01 | 0.438 | 0.750 |
| IC_50_ b12 | 0.500 | 1.000 |
| IC_50_ 2G12 | 1.000 | 1.000 |
| IC_50_ PGT121 | 0.635 | 0.386 |
| IC_50_ PGT128 | 0.563 | 1.000 |
| IC_50_ T20 | 0.563 | 0.250 |
| IC_50_ 2F5 | 0.313 | 0.500 |
| IC_50_ 4E10 | 0.875 | 0.500 |
| IC_50_ DARPin 27.2 | 0.563 | 0.250 |
| IC_50_ Maraviroc | 0.688 | 1.000 |
| Median AUC PBMCs | 0.156 | 1.000 |
| Median AUC MDMs | 0.844 | 0.500 |
| Median IFNα resistance | 0.156 | 0.250 |
| Median time to 50% entry | 0.438 | 0.250 |
| Cell-cell transmission | 0.219 | 0.250 |
| Free virus infection | 0.688 | 0.250 |

**Threshold for separation into high and low diversity transmitters 1.1%**

| Experiment | p value for high (T1-R1, T2-R2, T3-R3, T4-R4) | p value for low (T5-R5, T6-R6, T7-R7, T8-R8, T9-R9) |
| --- | --- | --- |
| V1V2 length | 1.000 | 1.000 |
| V1V2 glycosylation | 0.346 | not determined (same value) |
| V4 length | 1.000 | 0.500 |
| V4 glycosylation | 0.773 | 0.346 |
| Maximal percent neutralization transmitter plasma | not determined (only 2 values) | 0.813 |
| IC_50_ CD4**-**IgG2 | 0.250 | 0.125 |
| IC_50_ VRC01 | 0.875 | 0.375 |
| IC_50_ b12 | 0.500 | 1.000 |
| IC_50_ 2G12 | 1.000 | 0.750 |
| IC_50_ PGT121 | 0.660 | 0.732 |
| IC_50_ PGT128 | 1.000 | 0.375 |
| IC_50_ T20 | 0.375 | 0.438 |
| IC_50_ 2F5 | 0.500 | 0.125 |
| IC_50_ 4E10 | 0.750 | 0.875 |
| IC_50_ DARPin 27.2 | 0.875 | 0.125 |
| IC_50_ Maraviroc | 0.875 | 0.813 |
| Median AUC PBMCs | 0.250 | 0.813 |
| Median AUC MDMs | 0.625 | 0.125 |
| Median IFNα resistance | 0.250 | 0.063 |
| Median time to 50% entry | 0.250 | 0.313 |
| Cell-cell transmission | 0.250 | 0.188 |
| Free virus infection | 0.375 | 0.625 |

**Analysis for transmission pairs where recipient is closer to the MRCA**

| Experiment | p value close to MRCA (T1-R1, T2-R2, T3-R3, T6-R6, T7-R7, T9-R9) | p value (T4-R4, T5-R5, T8-R8) |
| --- | --- | --- |
| V1V2 length | 1.000 | 1.000 |
| V1V2 glycosylation | 1.000 | 1.000 |
| V4 length | 1.000 | 1.000 |
| V4 glycosylation | 0.233 | not determined (same value) |
| Maximal percent neutralization transmitter plasma | 0.625 | 0.500 |
| IC_50_ CD4**-**IgG2 | 1.000 | 0.500 |
| IC_50_ VRC01 | 0.563 | 0.500 |
| IC_50_ b12 | 1.000 | 1.000 |
| IC_50_ 2G12 | 0.500 | 0.500 |
| IC_50_ PGT121 | 0.813 | 0.500 |
| IC_50_ PGT128 | 1.000 | 0.250 |
| IC_50_ T20 | 0.688 | 0.250 |
| IC_50_ 2F5 | 0.219 | 1.000 |
| IC_50_ 4E10 | 0.438 | 0.500 |
| IC_50_ DARPin 27.2 | 0.313 | 0.500 |
| IC_50_ Maraviroc | 0.844 | 0.500 |
| Median AUC PBMCs | 0.156 | 1.000 |
| Median AUC MDMs | 0.313 | 0.750 |
| Median IFNα resistance | 0.156 | 0.250 |
| Median time to 50% entry | 0.094 | 1.000 |
| Cell-cell transmission | 0.156 | 0.500 |
| Free virus infection | 0.688 | 0.250 |

**Analysis for high diversity transmission pairs where recipient is closer to the MRCA**

| Experiment | p value (T1-R1, T2-R2, T3-R3, T9-R9) |
| --- | --- |
| V1V2 length | 1.000 |
| V1V2 glycosylation | 1.000 |
| V4 length | 1.000 |
| V4 glycosylation | 0.773 |
| Maximal percent neutralization transmitter plasma | not determined (2 values) |
| IC_50_ CD4**-**IgG2 | 0.500 |
| IC_50_ VRC01 | 0.625 |
| IC_50_ b12 | 1.000 |
| IC_50_ 2G12 | 1.000 |
| IC_50_ PGT121 | 0.875 |
| IC_50_ PGT128 | 1.000 |
| IC_50_ T20 | 0.875 |
| IC_50_ 2F5 | 0.375 |
| IC_50_ 4E10 | 1.000 |
| IC_50_ DARPin 27.2 | 0.875 |
| IC_50_ Maraviroc | 0.875 |
| Median AUC PBMCs | 0.125 |
| Median AUC MDMs | 0.875 |
| Median IFNα resistance | 0.375 |
| Median time to 50% entry | 0.375 |
| Cell-cell transmission | 0.375 |
| Free virus infection | 1.000 |
